# Depletion of Csk preferentially reduces the protein level of LynA in a Cbl-dependent manner in cancer cells

**DOI:** 10.1038/s41598-020-64624-x

**Published:** 2020-05-06

**Authors:** Takahisa Kuga, Yuka Yamane, Soujirou Hayashi, Masanari Taniguchi, Naoto Yamaguchi, Nobuyuki Yamagishi

**Affiliations:** 1 Laboratory of Analytics for Biomolecules, Faculty of Pharmaceutical Science, Setsunan University, Osaka, 573-0101 Japan; 2 Department of Molecular Cell Biology, Graduate School of Pharmaceutical Sciences, Chiba University, Chiba, 260-8675 Japan

**Keywords:** Molecular biology, Cell signalling

## Abstract

There are eight human Src-family tyrosine kinases (SFKs). SFK members c-Src, c-Yes, Fyn, and Lyn are expressed in various cancer cells. SFK kinase activity is negatively regulated by Csk tyrosine kinase. Reduced activity of Csk causes aberrant activation of SFKs, which can be degraded by a compensatory mechanism depending on Cbl-family ubiquitin ligases. We herein investigated whether all SFK members are similarly downregulated by Cbl-family ubiquitin ligases in cancer cells lacking Csk activity. We performed Western blotting of multiple cancer cells knocked down for Csk and found that the protein levels of the 56 kDa isoform of Lyn (LynA), 53 kDa isoform of Lyn (LynB), c-Src, and Fyn, but not of c-Yes, were reduced by Csk depletion. Induction of c-Cbl protein levels was also observed in Csk-depleted cells. The reduction of LynA accompanying the depletion of Csk was significantly reversed by the knockdown for Cbls, whereas such significant recovery of LynB, c-Src, and Fyn was not observed. These results suggested that LynA is selectively downregulated by Cbls in cancer cells lacking Csk activity.

## Introduction

The Src-family tyrosine kinases (SFKs) are composed of eight members in humans: c-Src, c-Yes, Fyn, Lyn, Lck, Fgr, Hck and Blk^1^. c-Src, c-Yes, Fyn and Lyn are widely expressed in a variety of cell types, whereas other members are primarily restricted to specific cell types^2^. The redundant expression of multiple members of SFKs is observed in almost all cell types^1^.

SFKs play important roles in various signalling pathways involved in various cellular events, including survival and proliferation, motility and invasion and angiogenesis^1,3,4^. The deregulated activation of SFKs promotes tumorigenesis and cancer progression^4,5^. The activity of SFKs has been reported to be associated with poor clinical prognosis of cancer patients^6,7^.

The kinase activity of SFKs is controlled by their post-translational modifications by phosphorylation. The autophosphorylation of the conserved tyrosine residue in their activation loop changes the protein structure into a fully active conformation^8,9^. In contrast, the phosphorylation of another conserved tyrosine residue in the C-terminal negative regulatory tail changes the protein structure into an inactive conformation^8,9^. This C-terminal tyrosine is phosphorylated by the Csk or Chk tyrosine kinases^10^. The loss of Csk therefore leads to the aberrant activation of SFKs^11,12^. The reduced activity of Csk is observed in hepatocellular carcinoma^13^.

The ubiquitin-proteasome system is another mechanism that down-regulates SFKs^14^. The degradation of SFKs by the ubiquitin-proteasome system serves as a mechanism to prevent aberrantly activated SFKs from excessively transducing signals. The ubiquitination-dependent degradation of c-Src and Fyn was observed in mouse embryonic fibroblasts containing a targeted disruption of the Csk gene^15^. The inhibition of Csk in bone marrow-derived macrophages led to the ubiquitination-dependent degradation of Lyn, Hck and Fgr^16^. The Cbl-family ubiquitin ligases composed of c-Cbl, Cbl-b and Cbl-c^17^ are involved in the ubiquitination of SFKs. At least c-Src, Fyn, Lyn and Lck were reported to be substrates of Cbls^18–23^.

We are interested in the results that the 56-kDa isoform of Lyn (LynA) is preferentially ubiquitinated and degraded compared to the 53-kDa isoform of Lyn (LynB), Hck and Fgr upon the inhibition of Csk in macrophages^16^. These results suggest that the ubiquitin-proteasome system functions with different selectivity against different members or splicing isoforms of SFKs in macrophages lacking the activity of Csk. In the present study, we examined whether particular members or splicing isoforms of SFKs were preferentially down-regulated by the protein degradation system when the activity of Csk was reduced in cancer cells where a different set of SFKs is expressed compared in macrophages.

## Results

### LynA protein levels are preferentially reduced in Csk-depleted cancer cells

**Figure 1:**
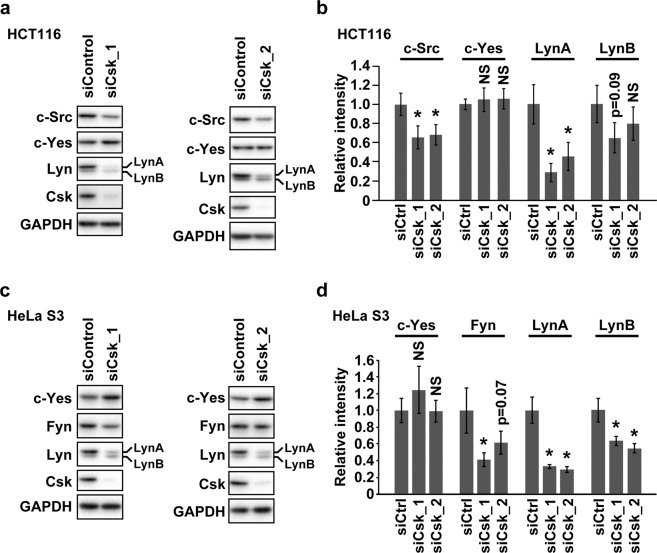
Depletion of Csk causes a preferential reduction of LynA. HCT116 (**a,b**) and HeLa S3 (**c,d**) cells were transfected with Csk siRNA or control siRNA and analyzed by Western blotting with antibodies against the indicated proteins. Two siRNAs for Csk with different nucleotide sequences were used. (**b,d**) show the average ± SD of the relative intensity of the individual bands calculated from three sets of samples (see also Fig. S1). The *p*-value against the control was calculated by Dunnett’s test. The asterisks and NS indicate *p* < 0.05 and *p* > 0.1, respectively. Full-length blots for (**a,c**) are presented in Fig. S7.

In order to assess the effect of reduced activity of Csk on the protein levels of individual members of SFKs, we performed Western blotting of two types of cancer cells (HCT116 and HeLa S3 cells) transfected with small interfering RNA (siRNA) for Csk (siCsk_1, SASI_Hs02_00328637, and siCsk_2, SASI_Hs02_00328637) or control siRNA. HCT116 cells redundantly expressed c-Src, c-Yes, and Lyn [Fig. 1(a)]. In HCT116 cells, the depletion of Csk remarkably reduced LynA and mildly reduced c-Src (Fig. 1(a,b), Fig. S1a). The protein level of LynB was slightly reduced upon the depletion of Csk in HCT116 cells, although this reduction was not statistically significant (Fig. 1(a,b), Fig. S1a). The protein level of c-Yes was not altered by the depletion of Csk in HCT116 cells (Fig. 1(a,b), Fig. S1a). HeLa S3 cells expressed c-Yes, Fyn, and Lyn [Fig. 1(c)]. HeLa S3 cells depleted of Csk exhibited reduced LynA, LynB, and Fyn levels (Fig. 1(c,d), Fig. S1b). The reduction level of LynA was higher than that of LynB (Fig. 1(d), Fig. S1b). Comparing the reduction level of Fyn with that of LynA or LynB was difficult because different reduction levels of Fyn were induced by two Csk siRNAs (Fig. 1(c,d), Fig. S1b). Csk depletion did not affect the c-Yes protein levels in HeLa S3 and HCT116 cells (Fig. 1(c,d), Fig. S1b). These results suggested that LynA protein levels are preferentially repressed via Csk depletion in cancer cells.

### Kinase activity of SFKs is required for the depletion of Csk to reduce the protein level of LynA

**Figure 2:**
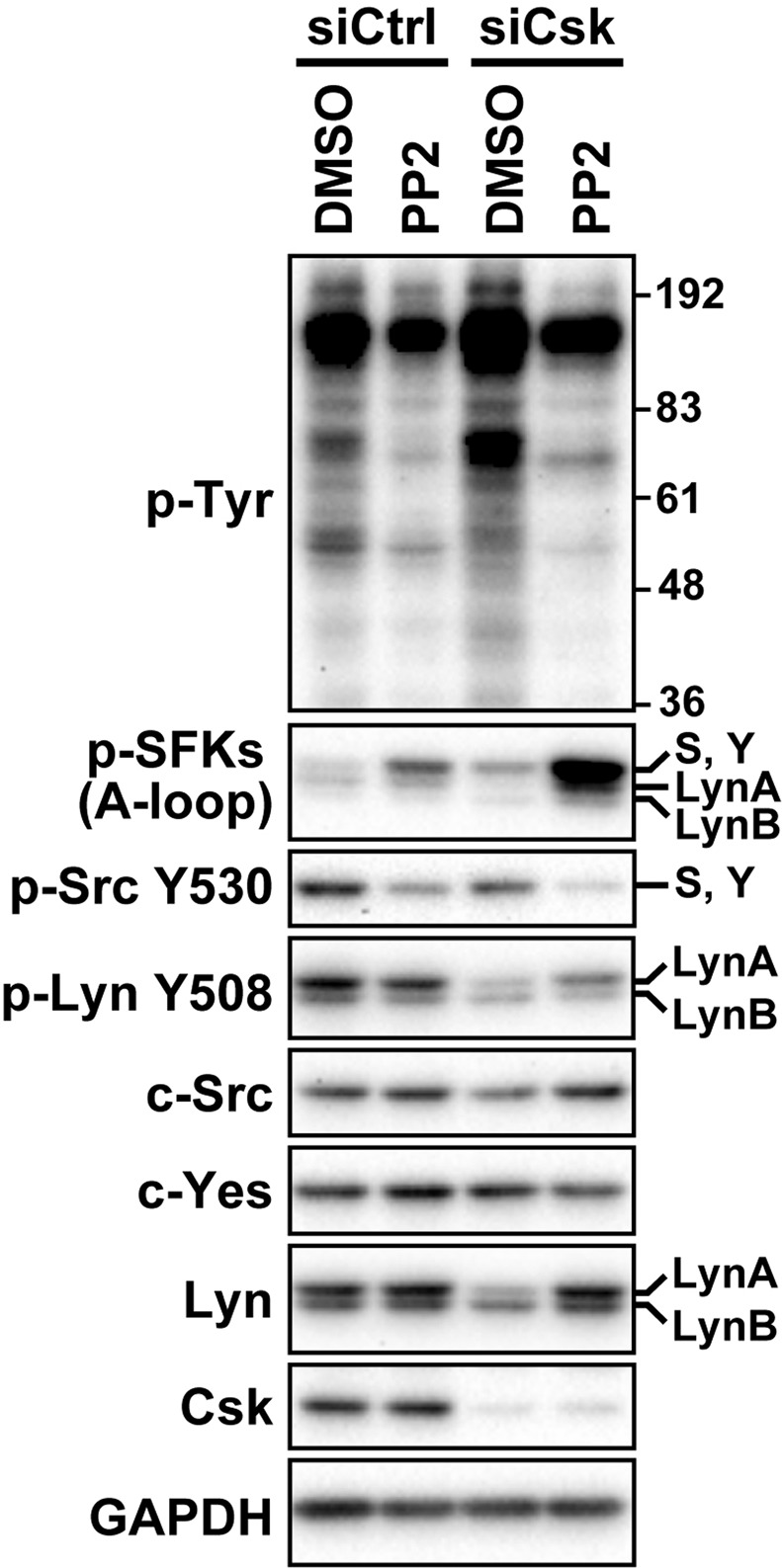
An inhibitor of SFKs, PP2, prevents the reduction of LynA accompanying the depletion of Csk. HCT116 cells were transfected with siRNA for Csk or the control siRNA and then cultured for 48 h. During the last 24 h of the total 48 h culture, cells were incubated in medium containing 10 μM PP2 or DMSO (solvent control) and analysed by Western blotting with antibodies against the indicated proteins. The numbers on the right side of the p-Tyr panel indicate the electrophoretic positions of the molecular weight marker proteins. The letters ‘S and Y’ on the right side of the panels for p-SFKs (A-loop) and p-Src Y530 indicate the electrophoretic position of c-Src and c-Yes. Full-length blots are presented in Fig. S8.

The depletion of Csk leads to the activation of SFKs by preventing the phosphorylation of the tyrosine residue at their C-terminal regulatory tail^8,9^. To determine whether the activation of SFKs participated in the process by which the depletion of Csk reduced LynA, we examined whether an SFK inhibitor, PP2^24^, was able to reverse the repressive effect of the depletion of Csk on the protein level of LynA. HCT116 cells were transfected with siRNA for Csk or control siRNA, and 24 h after transfection, these cells were treated with 10 μM PP2 or dimethyl sulfoxide (DMSO; control) for 24 h. These cells were analysed by Western blotting (Fig. 2). We first tried to confirm whether treatment with PP2 repressed the kinase activity of SFKs. In cells treated with PP2, the tyrosine phosphorylation levels at the activation loop and the C-terminal negative regulatory tail were not correlated to the kinase activity of SFKs^25^; thus, the effect of PP2 on the kinase activity of SFKs was not assessed by Western blotting with antibodies against phosphorylation at the conserved tyrosine in the activation loop [anti-p-SFKs (A-loop) antibody; Fig. 2] or in the C-terminal negative regulatory tail (anti-p-Src Y530 and anti-p-Lyn Y508 antibodies; Fig. 2). We therefore confirmed the effect of PP2 based on the reduction of the tyrosine phosphorylation of intracellular proteins detected by anti-phosphotyrosine (p-Tyr) antibody (Fig. 2, p-Tyr panel). In cells transfected with control siRNA, no influence of PP2 on the protein levels of SFKs was observed (Fig. 2, lanes 1 and 2). In cells depleted of Csk, treatment with PP2 reversed the reduction of LynA and also c-Src, whereas the protein level of c-Yes was comparable irrespective of whether cells treated with PP2 (Fig. 2, lanes 3 and 4). These results suggest that the activation of SFKs accompanying the depletion of Csk triggers the reduction of LynA.

### Constitutively active Src, v-Src, leads the reduction of LynA

**Figure 3:**
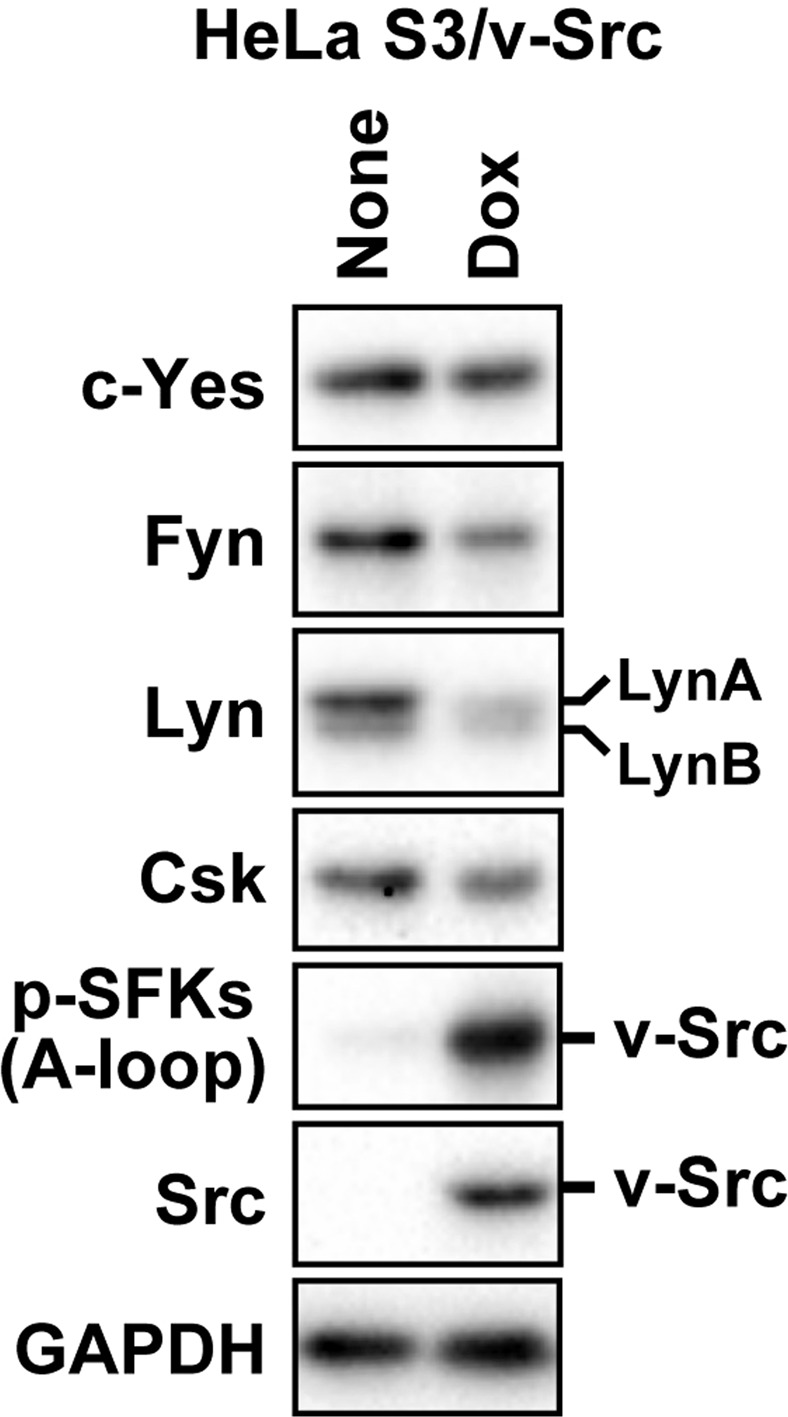
Ectopic expression of v-Src preferentially reduces LynA. HeLa S3/v-Src cells were treated or not with 2 ng/mL Dox for 6 h and then analysed by Western blotting with antibodies against the indicated proteins. Antibodies against Src or p-SFKs (A-loop) were used to confirm the expression of v-Src. Full-length blots are presented in Fig. S9.

To further verify whether the activation of SFKs leads the reduction of LynA, we examined whether constitutively active Src, v-Src^26^, leads the reduction of LynA. For this examination, we used HeLa S3/v-Src cells introduced with a system for the doxycycline (Dox)-inducible expression of v-Src^27^. HeLa S3/v-Src cells were treated or not with 2 ng/mL Dox for 6 h and analysed by Western blotting (Fig. 3). The Dox-induced expression of v-Src was confirmed using anti-Src and anti-p-SFKs (A-loop) antibodies. In cells expressing v-Src, the protein level of LynA was remarkably repressed. The protein levels of Fyn and LynB were mildly repressed. The protein level of c-Yes was not altered in cells expressing v-Src. Given that v-Src only slightly reduced the protein level of Csk, these results suggest that the aberrant activation of SFKs is sufficient to lead the reduction of LynA irrespective of whether Csk is depleted or not in cancer cells.

### Cbls are involved in Csk-mediated reduction of LynA

**Figure 4:**
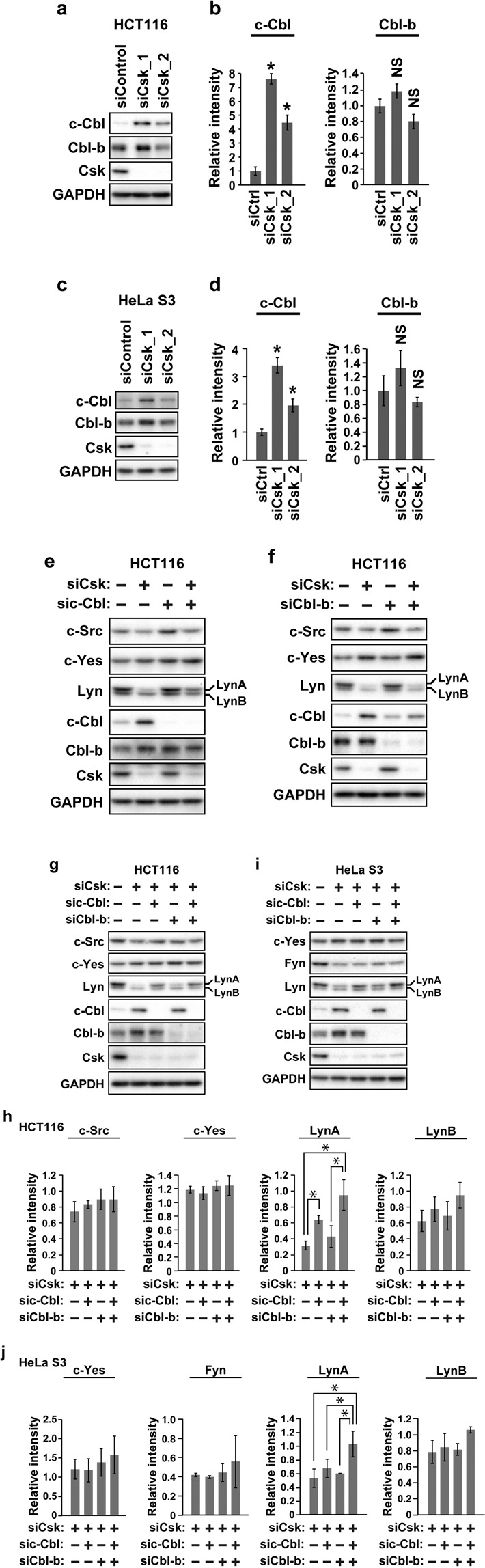
Cbls mediate the reduction of LynA accompanying the depletion of Csk. Western blot analysis with antibodies against the indicated proteins was conducted. HCT116 (**a,b**) or HeLa S3 (**c,d**) cells were transfected with siRNA for Csk or control siRNA and then cultured for 48 h. Two siRNAs for Csk with different nucleotide sequences were used. HCT116 (**e–h**) or HeLa S3 (**i,j**) cells were transfected with a combination of siRNAs for the indicated targets and cultured for 48 h. Control cells were transfected with control siRNA (e–g and i, each lane 1). Graphs (**b**), (**d**), (**h**), and (**j**) show the average ± SD of the relative intensity of the individual bands calculated from three sets of samples (see also Fig. S2). In graphs (**b**) and (**d**), the *p*-value against the control was calculated by Dunnett’s test, and the asterisks and NS indicate *p* < 0.05 and *p* > 0.1, respectively. In graphs (**h**) and (**j**), the *p*-values between all pairs of samples were calculated by Tukey’s HSD test. Significant differences (*p* < 0.05) were found only between the pairs indicated by the asterisks. Full-length blots are presented in Fig. S10.

A previous study showed that LynA was ubiquitinated by c-Cbl and subsequently degraded by the proteasome when mast cells were stimulated by FcεRI^22^. We assessed whether Cbls are involved in the process through which the depletion of Csk reduces LynA in cancer cells. We first examined whether the depletion of Csk affected the protein levels of c-Cbl and Cbl-b. Western blot analysis of HCT116 or HeLa S3 cells transfected with Csk siRNA showed that the depletion of Csk increased the protein level of c-Cbl, whereas the protein level of Cbl-b was not affected (Fig. 4(a–d), Fig. S2a,b). We then examined whether the knockdown of Cbls was able to reverse the downregulation of LynA in Csk-depleted cells. In Fig. 4(e–h), HCT116 cells were cotransfected with siRNAs for Csk, c-Cbl, and Cbl-b in the indicated combinations. The depletion of c-Cbl reversed the reduction of LynA (Fig. 4(e,g,h), Fig. S2c), whereas the depletion of Cbl-b did not cause a significant recovery of LynA (Fig. 4(f,h), Fig. S2c). The combined depletion of c-Cbl and Cbl-b strongly reversed the reduction of LynA compared to the single depletion of c-Cbl in HCT116 cells (Fig. 4(g,h), Fig. S2c; Tukey’s honestly significant difference [HSD] test; Fig. 4(h), LynA, lane 2 versus lane 4, *p* = 0.062). In HeLa S3 cells, the combined depletion of c-Cbl and Cbl-b significantly reversed the reduction of LynA accompanying the depletion of Csk, although the single depletion of c-Cbl or Cbl-b did not significantly affect the LynA levels (Fig. 4(i,J), Fig. S2d). We were not able to detect the significant recovery effect of the depletion of Cbls on the reduction of LynB, c-Src, and Fyn in Csk-depleted HCT116 and HeLa S3 cells, as judged by Tukey’s HSD statistical test (Fig. 4(e–j), Figs. S2c,d). The c-Yes protein levels were not altered by the depletion of Cbls in Csk-depleted HCT116 and HeLa S3 cells (Fig. 4(e–j), Figs. S2c,d). These results suggested that Cbls preferentially target LynA in Csk-depleted cancer cells.

### Induction of c-Cbl accompanying the depletion of Csk does not depend on the activation of SFKs

**Figure 5:**
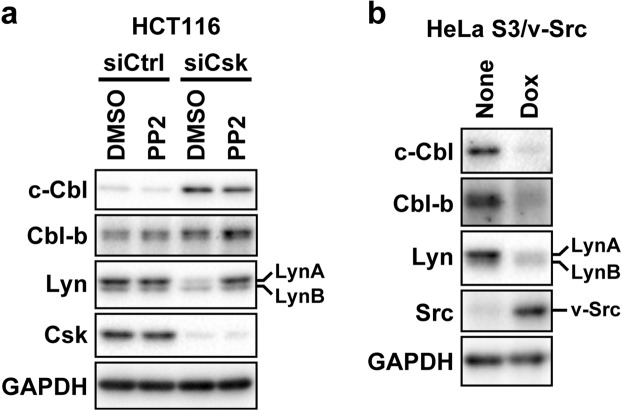
Activation of SFKs is not involved in the process by which the depletion of Csk increases the protein level of c-Cbl. Western blot analysis with antibodies against the indicated proteins. (**a**) Samples were prepared as described in Fig. 2(b). Samples were prepared as described in Fig. 3. Full-length blots are presented in Fig. S11.

As already described in Fig. 2, the reduction of LynA accompanying the depletion of Csk depended on the kinase activity of SFKs. To test whether the induction of c-Cbl accompanying the depletion of Csk also depends on the kinase activity of SFKs, we examined whether the induction of c-Cbl was reversed by treatment with PP2. Western blot analysis in Fig. 5a shows that the protein level of c-Cbl was comparably high irrespective of treatment with PP2 in HCT116 cells depleted of Csk. This result suggests that the activation of SFKs after the depletion of Csk is not required for the induction of c-Cbl. In HeLa S3 cells expressing v-Src, the protein levels of c-Cbl and Cbl-b were remarkably reduced (Fig. 5b); thus, the activation of SFKs seems to exert negative, rather than positive, impact on the protein levels of Cbls.

### LynA reduction accompanying Csk depletion does not affect epithelial or mesenchymal marker protein levels

**Figure 6:**
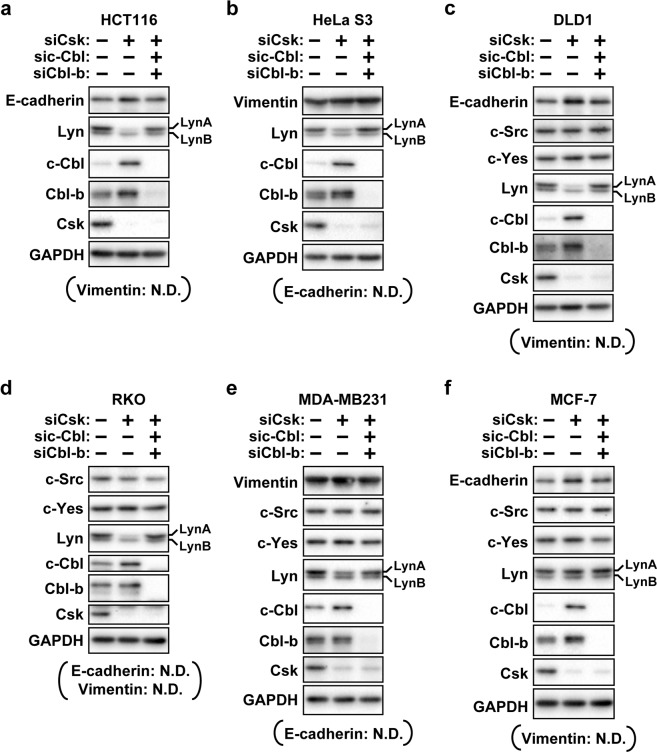
No relationships between the protein level of LynA and that of E-cadherin or vimentin. HCT116 (**a**), HeLa S3 (**b**), DLD1 (**c**), RKO (**d**), MDA-MB231 (**e**) and MCF-7 (**f**) cells were transfected with siRNA for Csk (lane 2), a combination of siRNAs for Csk, c-Cbl and Cbl-b (lane 3) or the control siRNA (lane 1) and then cultured for 48 h and analysed by Western blotting with antibodies against the indicated proteins. E-cadherin was not detected (N.D.) in HeLa S3, RKO and MDA-MB231 cells. Vimentin was N.D. in HCT116, DLD1, RKO and MCF-7 cells. Full-length blots are presented in Fig. S12.

LynA was reported to be a mediator of the epithelial–mesenchymal transition (EMT), decreasing and increasing epithelial and mesenchymal marker protein levels, respectively^28,29^. This knowledge led us to hypothesize that LynA reduction accompanying Csk depletion serves as a mechanism to protect cancer cells from undergoing EMT. In order to test this hypothesis, we performed Western blotting using antibodies against an epithelial marker protein, E-cadherin, and a mesenchymal marker protein, vimentin. Figure 6(a,b) show the Western blot analysis of HCT116 and HeLa S3 cells transfected with control siRNA, siRNA for Csk, or a combination of siRNAs for Csk, c-Cbl, and Cbl-b. In HCT116 cells, the protein levels of E-cadherin were almost comparable among the three samples [Fig. 6(a)], and vimentin was not detected in all three samples (data not shown). In HeLa S3 cells, the protein levels of vimentin were comparable among the three samples [Fig. 6(b)], and E-cadherin was not detected in all three samples (data not shown). These results suggested that the reduction of LynA accompanying the depletion of Csk does not affect the protein levels of E-cadherin and vimentin in HCT116 and HeLa S3 cells. We further performed similar experiments using DLD1 and RKO colorectal cancer cell lines and MDA-MB231 and MCF-7 breast cancer cell lines [Fig. 6(c–f)]. In DLD1, RKO, and MDA-MB231 cell lines, we observed reduced LynA protein levels accompanying Csk depletion and recovery from LynA reduction upon the codepletion of c-Cbl and Cbl-b [Fig. 6(c–e)]. In DLD1 cells, E-cadherin was detected in all three samples [Fig. 6(c)], whereas vimentin was not detected in all three samples (data not shown). In RKO cells, both E-cadherin and vimentin were not detected in all three samples (data not shown). In MDA-MB231 cells, the protein levels of vimentin were comparable among the three samples [Fig. 6(e)], and E-cadherin was not detected in all three samples (data not shown). These results did not show a dynamic influence of LynA reduction and Csk depletion on EMT marker protein levels. In MCF-7 cells, we observed that Csk depletion enhanced c-Cbl protein levels but did not cause LynA reduction [Fig. 6(f)]. This result indicated that the upregulation of c-Cbl is not sufficient for Csk depletion to reduce LynA in MCF-7 cells. Taken together, our data suggested that LynA reduction accompanying Csk depletion does not serve as a mechanism to protect cancer cells from undergoing EMT. In future studies, we will try to elucidate the biological and physiological significance of LynA reduction in cancer cells lacking Csk activity.

## Discussion

A previous study showed that LynA was preferentially degraded, compared to LynB, Hck and Fgr, when Csk was inhibited in macrophages^16^. In the present study, we examined the influence of the depletion of Csk on the protein levels of SFK members in cancer cells, which express a different set of SFK members compared to macrophages. We demonstrated that the aberrant activation of SFKs accompanying the depletion of Csk leads the preferential reduction of LynA, compared at least to c-Src, c-Yes and LynB, in a manner depending on Cbls in multiple epithelial cancer cells. Given the knowledge that LynA drives migration and invasion in breast cancer cells^30^, our results may suggest that the preferential reduction of LynA is a feedback mechanism for preventing cancer progression in cancer cells lacking the activity of Csk.

The requirement of Cbls in depleted Csk-mediated reduction of LynA in cancer cells (Figs. 4 and 6) indicates the role of the ubiquitin–proteasome system in LynA reduction. In macrophages, the degradation of LynA accompanying the inhibition of Csk was demonstrated to be mediated by the ubiquitin–proteasome system, although the involvement of Cbls was not tested^16^. Previous reports showed that Cbls can bind to and ubiquitinate Lyn. In RBL-2H3 mast cells, the stimulation of the high-affinity IgE receptor (FcεRI) resulted in the association of Lyn with c-Cbl and Cbl-b and subsequent ubiquitination of Lyn^22^. The pull-down assay using GST-fused N-terminal regions of Lyn also supported the association of Lyn with c-Cbl^31,32^. Ubiquitination of Lyn was induced by the overexpression of c-Cbl in RBL-2H3 mast cells^22^. These previous studies suggested that LynA is ubiquitinated by Cbls and subsequently degraded by the proteasome in immune cells lacking Csk activity. Our results suggested that this mechanism for reducing LynA in response to reduced Csk activity is commonly used not only in immune cells, but also in cancer cells, though not all cancer cells. At least in MCF-7 cells, the reduced protein level of LynA was not induced by the depletion of Csk [Fig. 6(f)]. We have not discerned the reason for the inability of MCF-7 cells to downregulate LynA after the depletion of Csk.

Freedman *et al*. posited the following reason for the observed preferential reduction of LynA compared to LynB: “LynA differs from LynB only in a 21 amino-acid insert in its unique region, and this unique insert contains a predicted ubiquitination site (K40)”^16^. LynA is speculated to be more susceptible to ubiquitination and subsequent proteolysis compared to LynB. We further searched predicted ubiquitination sites in the SFK members using the UbPred program^33^. The predicted ubiquitination sites were present in all SFK members showing reduced protein levels after Csk depletion, that is, LynA (Lys20 and Lys40), LynB (Lys20), c-Src (Lys40), and Fyn (Lys13), whereas no predicted ubiquitination site was detected in c-Yes whose protein level is not reduced after the depletion of Csk (Fig. 1). We speculated that the primary structure of c-Yes might not be targeted by ubiquitin ligases such as Cbls.

Unexpectedly, the reduction of c-Src and Fyn after the depletion of Csk was not reversed by the depletion of Cbls in cancer cells [Fig. 4(g–j)]. This suggested that c-Src and Fyn, in contrast to LynA, are not under the control of Cbls in cancer cells depleted of Csk. On the other hand, it has been reported in a number of studies that c-Src and Fyn, as well as Lyn, can be targeted by Cbls for ubiquitination and subsequent degradation^18–20,22,23^; thus, selective targeting of Cbls to LynA may occur in limited situations, including under the condition in which Csk is depleted. Thus far, the mechanism of the reduction of c-Src and Fyn in cancer cells depleted of Csk is unclear. However, some questions still remain: (1) Is transcription or translation suppressed? (2) Is the degradation dependent on or independent of the ubiquitin–proteasome system? In order to assess the involvement of the ubiquitin–proteasome system in this mechanism, we attempted to examine whether the reduction of c-Src and Fyn after the depletion of Csk was reversed by a proteasome inhibitor, MG132, in HCT116 or HeLa S3 cells; however, we were not able to obtain convincing results because treatment with MG132 led to severe cell damage and death (data not shown).

Our results suggest that, to reduce LynA, the depletion of Csk accelerates the functions of Cbls by two mechanisms. Figure 2 reveals that one of the two mechanisms depends on the activation of SFKs accompanying the depletion of Csk (Fig. 2). A number of reports have shown that the kinase activity of SFKs is required for their association with Cbls^23,34^ and their ubiquitination by Cbls^18,21,23,34^. Furthermore, SFKs have been suggested to phosphorylate Tyr371 of c-Cbl, which corresponds to Tyr363 of Cbl-b^18^. Phosphorylation at this conserved tyrosine has been suggested to increase the E3 activity of Cbls^35,36^. Crystal structural analyses of the N-terminal region of Cbls have revealed that phosphorylation at this conserved tyrosine changes the conformation of Cbls from a closed, inactive state into an open, active state^37–39^. Although we have speculated that the depletion of Csk induces phosphorylation at this conserved tyrosine of Cbls, we have not yet tested this possibility because there is no commercially available antibody against Cbls that is phosphorylated at this conserved tyrosine.

Another mechanism by which the depletion of Csk accelerates the function of Cbls is the elevation of the protein level of c-Cbl (Figs. 4 and 6). Figure 5(a) suggests that this mechanism is not mediated by the activation of SFKs. Figure 5(b) suggests that the activation of SFKs rather decreases the protein level of c-Cbl. It has been suggested in previous studies that the activation of SFKs leads to not only the ubiquitination of SFKs, but also the self-ubiquitination and subsequent degradation of c-Cbl^18,40^. These results suggested that the depletion of Csk seems to promote intracellular activity to increase the protein level of c-Cbl that overcomes the activity of aberrantly activated SFKs to decrease c-Cbl. Thus far, we tested the involvement of protein kinase C (PKC) and transcription factor SP1 in this upregulation mechanism of c-Cbl, because previous studies indicated the involvement of these proteins in the regulation of the protein level of c-Cbl. Jeschke *et al*. showed that treatment of human preosteoclastic cells with a PKC activator, phorbol 12-myristate 13-acetate, enhanced the protein level of c-Cbl^41^. However, the involvement of PKC in the induction of c-Cbl accompanying the depletion of Csk may not be true, because the PKC inhibitor Gö6983^42^ (1 μM) did not reverse the induction of c-Cbl (Fig. S3). Wei *et al*. showed that the inhibition or knockdown of HDACs induced the expression of c-Cbl, and this induction was prevented by an SP1 inhibitor, mithramycin A^43^. We tested whether 1 μM mithramycin A prevented the depletion of Csk from increasing the protein level of c-Cbl in HCT116 cells. Mithramycin A decreased the protein level of c-Cbl irrespective of the depletion of Csk (Fig. S4). Although this result may suggest the involvement of SP1 in the regulation of the protein level of c-Cbl, we cannot determine from this result whether the depletion of Csk promotes SP1 to increase the protein level of c-Cbl.

Although the expression of v-Src reduced LynA (Fig. 3), this result does not mean that the activation of c-Src reduced LynA. Overexpression of HA-tagged c-Src in HeLa S3 cells did not reduce LynA (Fig. S5). Knockdown of c-Src, c-Yes, or their combination did not prevent the reduction of LynA accompanying the depletion of Csk (Fig. S6). These results indicated that the activation of c-Src and c-Yes do not mediate the reduction of LynA in cells depleted of Csk. Activation of LynA itself may be required to trigger its own reduction after Csk depletion.

Previous studies suggested that LynA seems to contribute to the aggressive behaviour of some types of cancer^28–30^. Tornillo *et al*. reported that LynA promotes tumour cell invasion and that a high LynA/LynB isoform ratio is associated with poor prognosis of breast cancer patients^30^. Two previous studies from different groups showed that Lyn mediates EMT^28,29^. Although the authors of these two studies did not mention which contributes to the induction of EMT (whether LynA or LynB), at least one study showed results suggesting that the overexpression of LynA induced EMT^28^. Given these results, the reduction of LynA may serve as a mechanism preventing aggressive behaviours such as invasion of cancer cells when the functions of Csk are compromised. However, this hypothesis was not supported by our results that the reduction of LynA was not associated with the reduction of E-cadherin or the induction of vimentin in cells depleted of Csk (Fig. 6). We need further studies to demonstrate the physiological importance of the preferential reduction of LynA in cancer cells lacking the activity of Csk.

## Methods

### Cell culture and transfection

HCT116 and RKO colorectal cancer cells and MDA-MB231 and MCF-7 breast cancer cells were obtained from the American Type Culture Collection (Manassas, VA, USA). HeLa S3 cervical cancer cells were obtained from the Japanese Collection of Research Bioresources (Osaka, Japan). DLD1 colorectal cancer cells were obtained as described previously^44^. HeLa S3/v-Src cells capable of the inducible expression of v-Src were previously generated^27^. HeLa S3/c-Src-HA cells capable of the inducible expression of C-terminal HA-tagged c-Src were previously generated^45^. All cell lines were cultured at 37 °C in 5% CO_2_ in Iscove’s modified Dulbecco’s medium (Sigma-Aldrich, St. Louis, MO, USA) supplemented with 5% foetal bovine serum (PAA Laboratories GmbH, Pasching, Austria). Dox (Sigma-Aldrich), PP2 (Calbiochem, La Jolla, CA, USA), Gö6983 (Cyman Chemical, Ann Arbor, MI, USA) and mithramycin A (Cyman Chemical) were purchased from some vendors. Transfection with siRNAs was performed using Lipofectamine RNAiMAX (Life Technologies, Carlsbad, CA, USA). All siRNAs were purchased from Sigma-Aldrich: Mission SIC-001 as control siRNA, SASI_Hs02_00328637 (siCsk_1) and SASI_Hs02_00328637 (siCsk_2) for Csk, SASA_Hs01_00095408 for c-Cbl, SASI_Hs02_00367566 for Cbl-b, SASI_Hs01_00115730 for c-Src, SASI_Hs01_00086923 for c-Yes and SASI_Hs02_00325236 for Lyn. Except for the cases especially mentioned, siCsk_1 was used for the knockdown for Csk.

### Antibodies

The following antibodies were used: anti-Src (05–184; Merck, Darmstadt, Germany), anti-Yes (610375; BD Biosciences, San Jose, CA, USA), anti-Lyn (sc-7274; Santa Cruz Biotechnology, Dallas, TX, USA), anti-Csk (610080; BD Biosciences), anti-glyceraldehyde 3-phosphate dehydrogenase (GAPDH) (2275-PC-100; R&D Systems, Minneapolis, MN, USA), anti-p-Tyr (05–1050; Merck), anti-Src family phospho-Y418 (p-SFKs A-loop; ab40660; Abcam, Cambridge, UK), anti-p-Src Y530 (sc-166860; Santa Cruz Biotechnology), anti-p-Lyn Y508 (CSB-PA000691; Cusabio, Wuhan, China)), anti-Fyn (sc-434; Santa Cruz Biotechnology), anti-E-cadherin (#3195; Cell Signaling Technology, Danvers, MA, USA), anti-vimentin (V6630; Sigma-Aldrich), anti-Cbl-b (sc-8006; Santa Cruz Biotechnology) and anti-c-Cbl (sc-1651; Santa Cruz Biotechnology). Horseradish peroxidase-conjugated anti-mouse IgG (#7076; Cell Signaling Technology) and anti-rabbit IgG (711-035-152; Jackson Immunoresearch, West Grove, PA, USA) antibodies were used for Western blotting.

### Protein extraction and Western blotting

For protein extraction, cells were directly lysed in sodium dodecyl sulphate-polyacrylamide gel electrophoresis sample buffer. Western blotting was performed using Clarity Western ECL Substrate (Bio-Rad, Hercules, CA, USA) or Chemi-Lumi One Ultra (Nacalai Tesque, Kyoto, Japan). Images were obtained with ChemiDoc MP (Bio-Rad) and processed with Photoshop CS5 (Adobe, San Jose, CA, USA). Quantitation of the band intensity was performed using the ImageJ software. Statistical analysis was performed using the JMP software (SAS Institute Inc., Cary, NC, USA).

## Supplementary information

Supplementary information.
