# Supplementary material for: Depletion of Csk preferentially reduces the protein level of LynA in a Cbl-dependent manner in cancer cells
Source: Sci Rep. 2020 May 6;10:7621. doi: 10.1038/s41598-020-64624-x (PMC7203244; doi:10.1038/s41598-020-64624-x)
Supplement: Supplementary file 1 — Supplementary information. [file 41598_2020_64624_MOESM1_ESM.pdf]

Depletion of Csk preferentially reduces the protein level of LynA in a Cbl-dependent manner in cancer cells

Takahisa Kuga, Yuka Yamane, Soujirou Hayashi, Masanari Taniguchi, Naoto Yamaguchi and Nobuyuki Yamagishi

Supplementary figure legends

Fig. S1 Western blotting for quantitation in Figs. 1(b) and 1(d).

Samples were prepared as described in Fig. 1. Blot images for the indicated proteins are shown. (a) and (b) correspond to Figs. 1(b) and 1(d), respectively. The relative intensities of the bands are indicated by asterisks, shown under each panel. The average value of the three control samples is set to "1.00." The quantitative value normalized by the value for GAPDH is used in Figs. 1(b) and 1(d). The numbers on the right-hand side of each panel indicate the electrophoretic positions of the molecular weight marker proteins.

Fig. S2 Western blotting for quantitation in Figs. 4(b), 4(d), 4(h), and 4(j).

Samples were prepared as described in Fig. 4. Blot images for the indicated proteins are shown. (a), (b), (c), and (d) correspond to Figs. 4(b), 4(d), 4(h), and 4(j), respectively. The relative intensities of the bands are indicated by asterisks, shown under each panel. In (a) and (b), the average value of the three control samples is set to "1.00." In (c) and (d), the value for the control of each sample set is set to "1.00." The quantitative value normalized by the value for GAPDH is used in Figs. 4(b), 4(d), 4(h), and 4(j). The numbers on the right-hand side of each panel indicate the electrophoretic positions of the molecular weight marker proteins.

Fig. S3 A PKC inhibitor, Gö6983, did not reverse the induction of c-Cbl accompanying the depletion of Csk.

HCT116 cells were transfected with siRNA for Csk or the control siRNA, and 24 h after transfection, treated with 1 μM Gö6983 or the solvent control (DMSO) for 24 h. These cells were analyzed by Western blotting with antibodies against the indicated proteins. (a) Cropped images. (b) Full-length images.

Fig. S4 Effects of a SP1 inhibitor, mithramycin A, on the protein levels of Cbls.

HCT116 cells were transfected with siRNA for Csk or the control siRNA, and 24 h after transfection, treated with 1 μM mithramycin A or the solvent control (DMSO) for 24 h. These cells were analyzed by Western blotting with antibodies against the indicated proteins. (a) Cropped images. (b) Full-length images.

Fig. S5 Overexpression of c-Src did not reduce LynA.

HeLa S3/c-Src-HA cells were treated with 1 mM Dox for 24 h and analyzed by Western blotting with antibodies against the indicated proteins. Anti-p-SFKs (A-loop) and anti-Src antibodies were used for the confirmation of the expression of c-Src-HA. (a) Cropped images. (b) Full-length images.

Fig. S6 Depletion of c-Src and c-Yes did not reverse the reduction of LynA accompanying the depletion of Csk.

HCT116 cells were transfected with a combination of the indicated siRNAs and cultured for 48 h. These cells were analyzed by Western blotting with antibodies against the indicated proteins. The control cells were transfected with the control siRNA (each lane 1). (a-c) Cropped images. (d-f) Full-length images included in (a-c), respectively.

Fig. S7 Full-length images for Western blotting included in Fig. 1.

(a) and (b) correspond to Fig. 1a and c, respectively.

Fig. S8 Full-length images for Western blotting included in Fig. 2.

Fig. S9 Full-length images for Western blotting included in Fig. 3.

Fig. S10 Full-length images for Western blotting included in Fig. 4

(a), (b), (c), (d), (e) and (f) correspond to Fig. 4a, c, e, f, g and i, respectively.

Fig. S11 Full-length images for Western blotting included in Fig. 5.

(a) and (b) correspond to Fig. 5a and b, respectively.

Fig. S12 Full-length images for Western blotting included in Fig. 6.

(a), (b), (c), (d), (e) and (f) correspond to Fig. 6a, b, c, d, e and f, respectively.

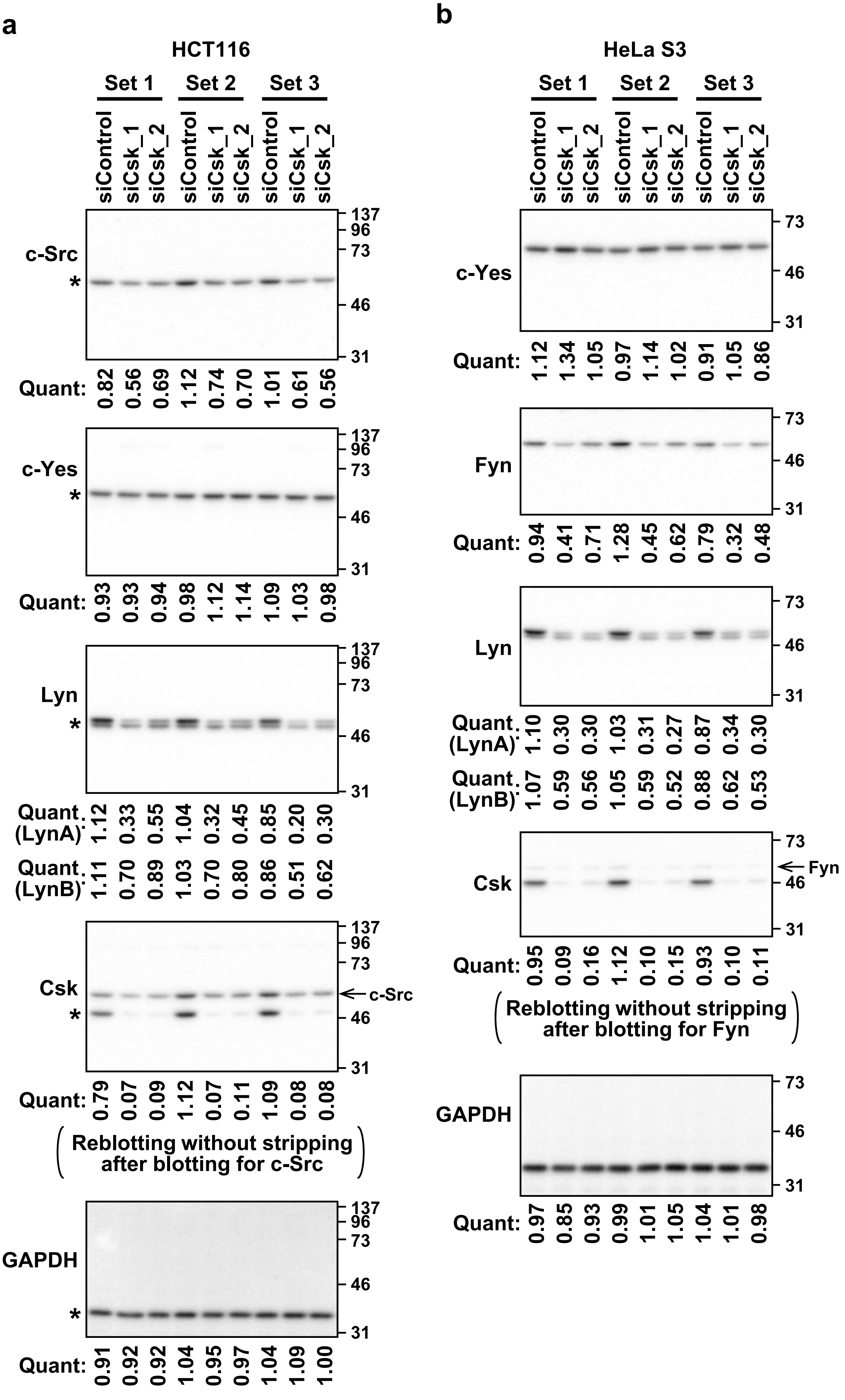
a
HCT116
Set 1
Set 2
Set 3
siControl
siCsk_1
siCsk_2
c-Src
137
96
73
46
31
Quant: 0.82 0.56 0.69 1.12 0.74 0.70 1.01 0.61 0.56
c-Yes
Quant: 0.93 0.93 0.94 0.98 1.12 1.14 1.09 1.03 0.98
Lyn
Quant (LynA): 1.12 0.33 0.55 1.04 0.32 0.45 0.85 0.20 0.30
Quant (LynB): 1.11 0.70 0.89 1.03 0.70 0.80 0.86 0.51 0.62
Csk
c-Src
Quant: 0.79 0.07 0.09 1.12 0.07 0.11 1.09 0.08 0.08
(Reblotting without stripping after blotting for c-Src)
GAPDH
Quant: 0.91 0.92 0.92 1.04 0.95 0.97 1.04 1.09 1.00
b
HeLa S3
c-Yes
Quant: 1.12 1.34 1.05 0.97 1.14 1.02 0.91 1.05 0.86
Fyn
Quant: 0.94 0.41 0.71 1.28 0.45 0.62 0.79 0.32 0.48
Lyn
Quant (LynA): 1.10 0.30 0.30 1.03 0.31 0.27 0.87 0.34 0.30
Quant (LynB): 1.07 0.59 0.56 1.05 0.59 0.52 0.88 0.62 0.53
Csk
Fyn
Quant: 0.95 0.09 0.16 1.12 0.10 0.15 0.93 0.10 0.11
(Reblotting without stripping after blotting for Fyn)
GAPDH
Quant: 0.97 0.85 0.93 0.99 1.01 1.05 1.04 1.01 0.98

Figure S1

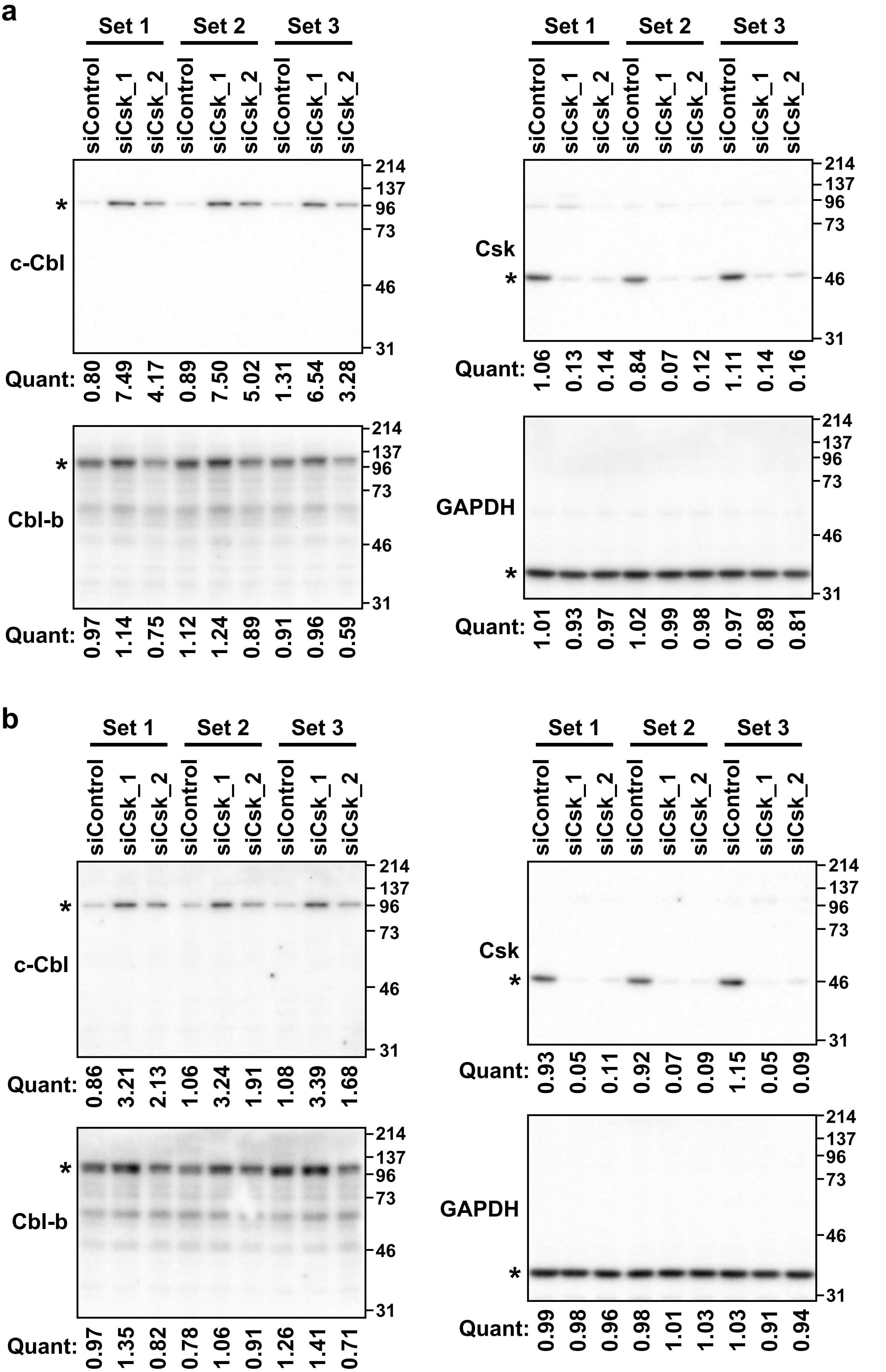
a
Set 1
Set 2
Set 3
siControl
siCsk_1
siCsk_2
c-Cbl
Quant: 0.80 7.49 4.17 0.89 7.50 5.02 1.31 6.54 3.28
Cbl-b
Quant: 0.97 1.14 0.75 1.12 1.24 0.89 0.91 0.96 0.59
Csk
Quant: 1.06 0.13 0.14 0.84 0.07 0.12 1.11 0.14 0.16
GAPDH
Quant: 1.01 0.93 0.97 1.02 0.99 0.98 0.97 0.89 0.81
214
137
96
73
46
31
b
c-Cbl
Quant: 0.86 3.21 2.13 1.06 3.24 1.91 1.08 3.39 1.68
Cbl-b
Quant: 0.97 1.35 0.82 0.78 1.06 0.91 1.26 1.41 0.71
Csk
Quant: 0.93 0.05 0.11 0.92 0.07 0.09 1.15 0.05 0.09
GAPDH
Quant: 0.99 0.98 0.96 0.98 1.01 1.03 1.03 0.91 0.94

Figure S2-1

c

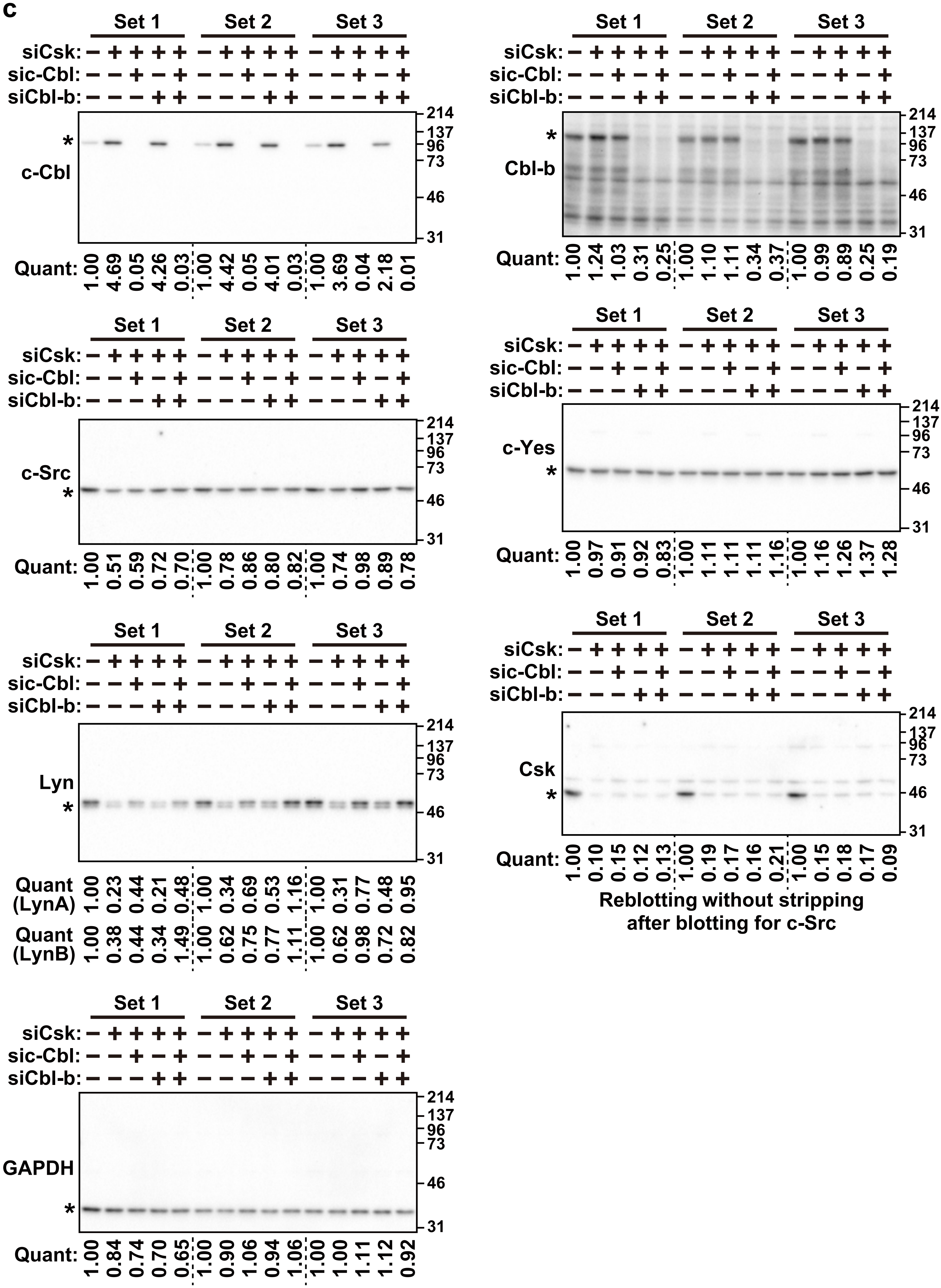
Set 1
Set 2
Set 3
siCsk:
sic-Cbl:
siCbl-b:
c-Cbl
Quant: 1.00 4.69 0.05 4.26 0.03 1.00 4.42 0.05 4.01 0.03 1.00 3.69 0.04 2.18 0.01
Cbl-b
Quant: 1.00 1.24 1.03 0.31 0.25 1.00 1.10 1.11 0.34 0.37 1.00 0.99 0.89 0.25 0.19
c-Src
Quant: 1.00 0.51 0.59 0.72 0.70 1.00 0.78 0.86 0.80 0.82 1.00 0.74 0.98 0.89 0.78
c-Yes
Quant: 1.00 0.97 0.91 0.92 0.83 1.00 1.11 1.11 1.11 1.16 1.00 1.16 1.26 1.37 1.28
Lyn
Quant (LynA) 1.00 0.23 0.44 0.21 0.48 1.00 0.34 0.69 0.53 1.16 1.00 0.31 0.77 0.48 0.95
Quant (LynB) 1.00 0.38 0.44 0.34 1.49 1.00 0.62 0.75 0.77 1.11 1.00 0.62 0.98 0.72 0.82
Csk
Quant: 1.00 0.10 0.15 0.12 0.13 1.00 0.19 0.17 0.16 0.21 1.00 0.15 0.18 0.17 0.09
Reblotting without stripping after blotting for c-Src
GAPDH
Quant: 1.00 0.84 0.74 0.70 0.65 1.00 0.90 1.06 0.94 1.06 1.00 1.00 1.11 1.12 0.92
214
137
96
73
46
31

Figure S2-2

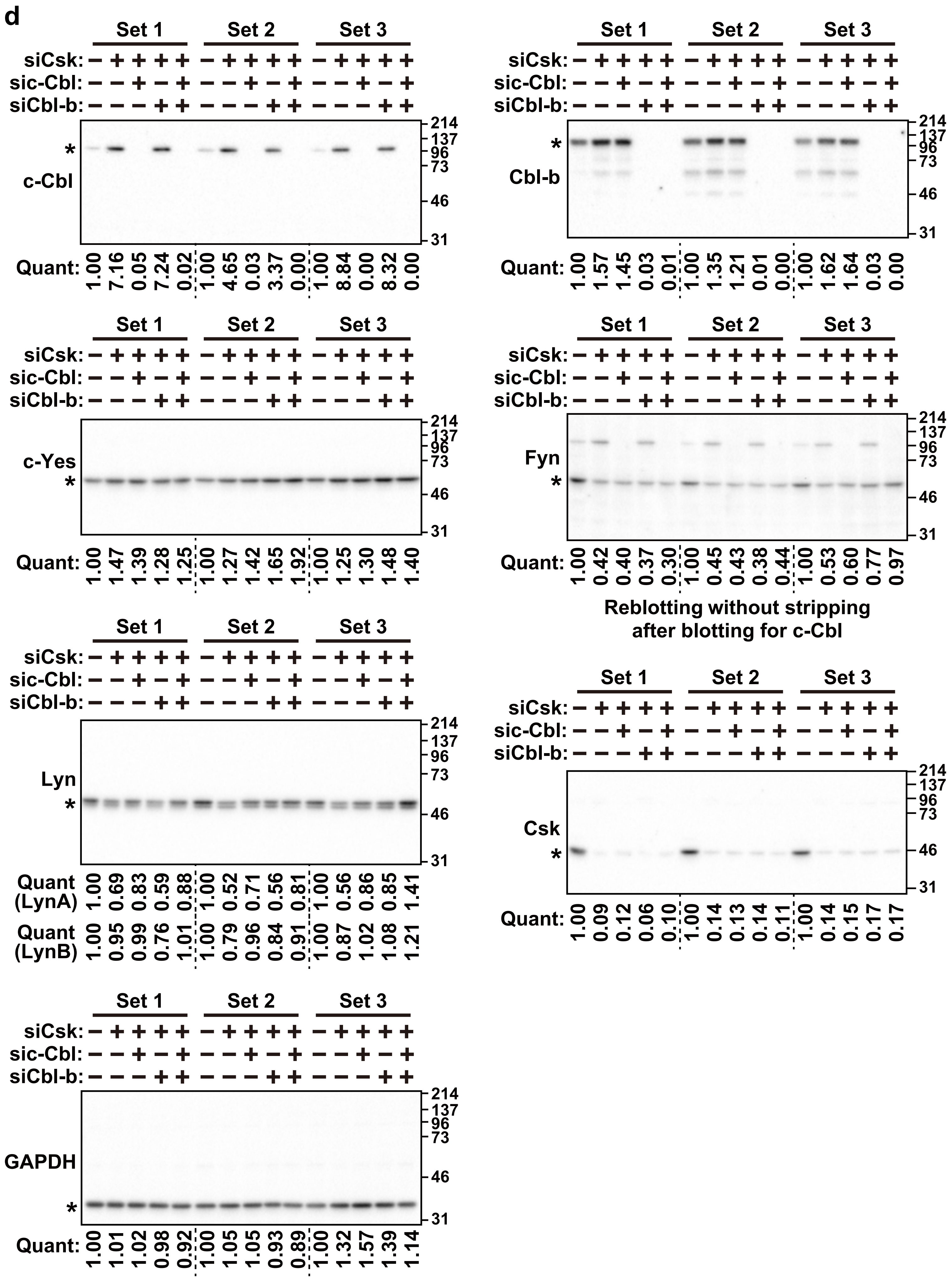
d
Set 1
Set 2
Set 3
siCsk:
sic-Cbl:
siCbl-b:
*
c-Cbl
214
137
96
73
46
31
Quant: 1.00 7.16 0.05 7.24 0.02 1.00 4.65 0.03 3.37 0.00 1.00 8.84 0.00 8.32 0.00
Cbl-b
Quant: 1.00 1.57 1.45 0.03 0.01 1.00 1.35 1.21 0.01 0.00 1.00 1.62 1.64 0.03 0.00
c-Yes
Quant: 1.00 1.47 1.39 1.28 1.25 1.00 1.27 1.42 1.65 1.92 1.00 1.25 1.30 1.48 1.40
Fyn
Quant: 1.00 0.42 0.40 0.37 0.30 1.00 0.45 0.43 0.38 0.44 1.00 0.53 0.60 0.77 0.97
Reblotting without stripping
after blotting for c-Cbl
Lyn
Quant (LynA) 1.00 0.69 0.83 0.59 0.88 1.00 0.52 0.71 0.56 0.81 1.00 0.56 0.86 0.85 1.41
Quant (LynB) 1.00 0.95 0.99 0.76 1.01 1.00 0.79 0.96 0.84 0.91 1.00 0.87 1.02 1.08 1.21
Csk
Quant: 1.00 0.09 0.12 0.06 0.10 1.00 0.14 0.13 0.14 0.11 1.00 0.14 0.15 0.17 0.17
GAPDH
Quant: 1.00 1.01 1.02 0.98 0.92 1.00 1.05 1.05 0.93 0.89 1.00 1.32 1.57 1.39 1.14

Figure S2-3

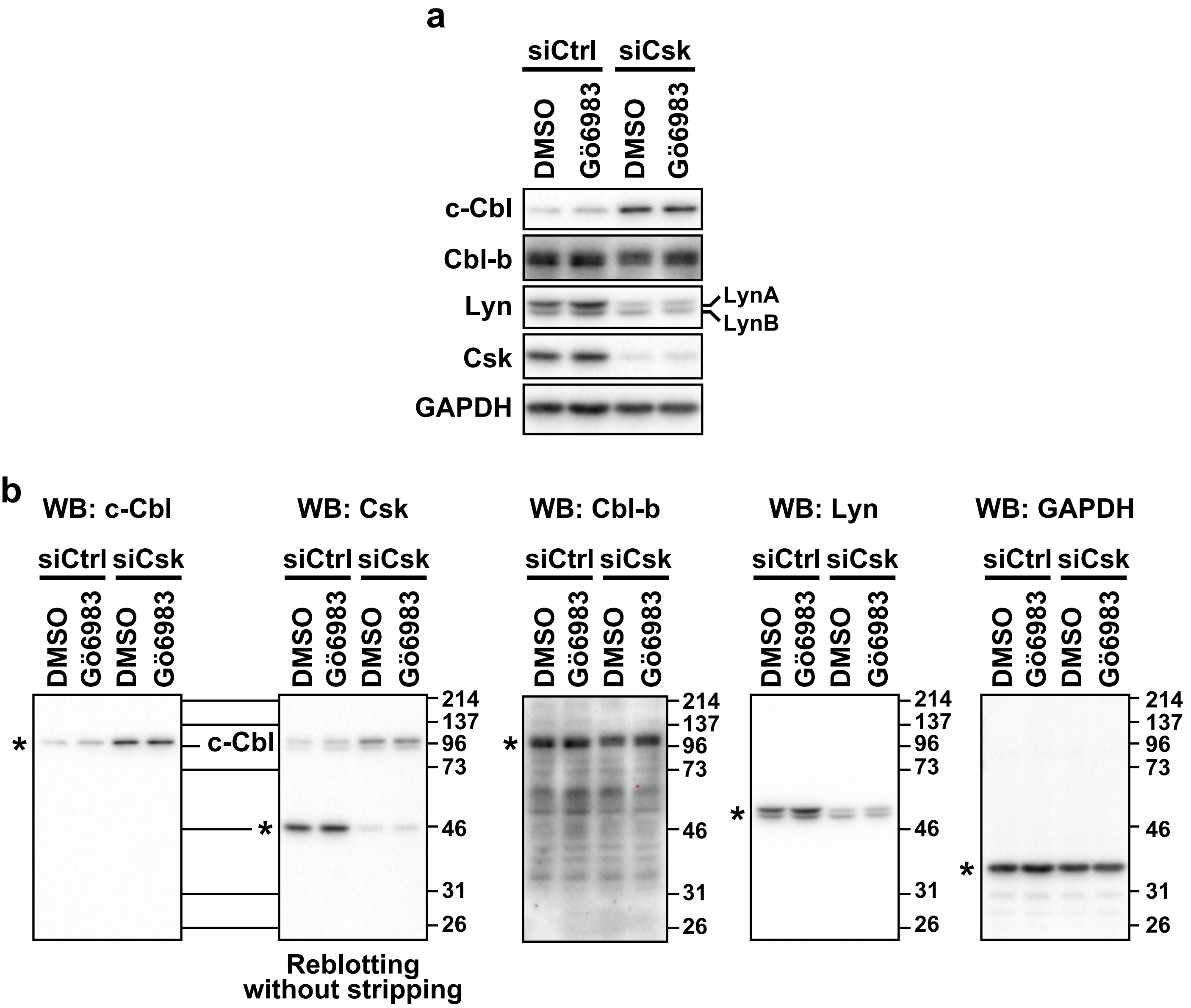
a
siCtrl
siCsk
DMSO
Gö6983
DMSO
Gö6983
c-Cbl
Cbl-b
Lyn
LynA
LynB
Csk
GAPDH
b
WB: c-Cbl
WB: Csk
WB: Cbl-b
WB: Lyn
WB: GAPDH
siCtrl
siCsk
DMSO
Gö6983
DMSO
Gö6983
*
c-Cbl
214
137
96
73
46
31
26
Reblotting
without stripping

Figure S3

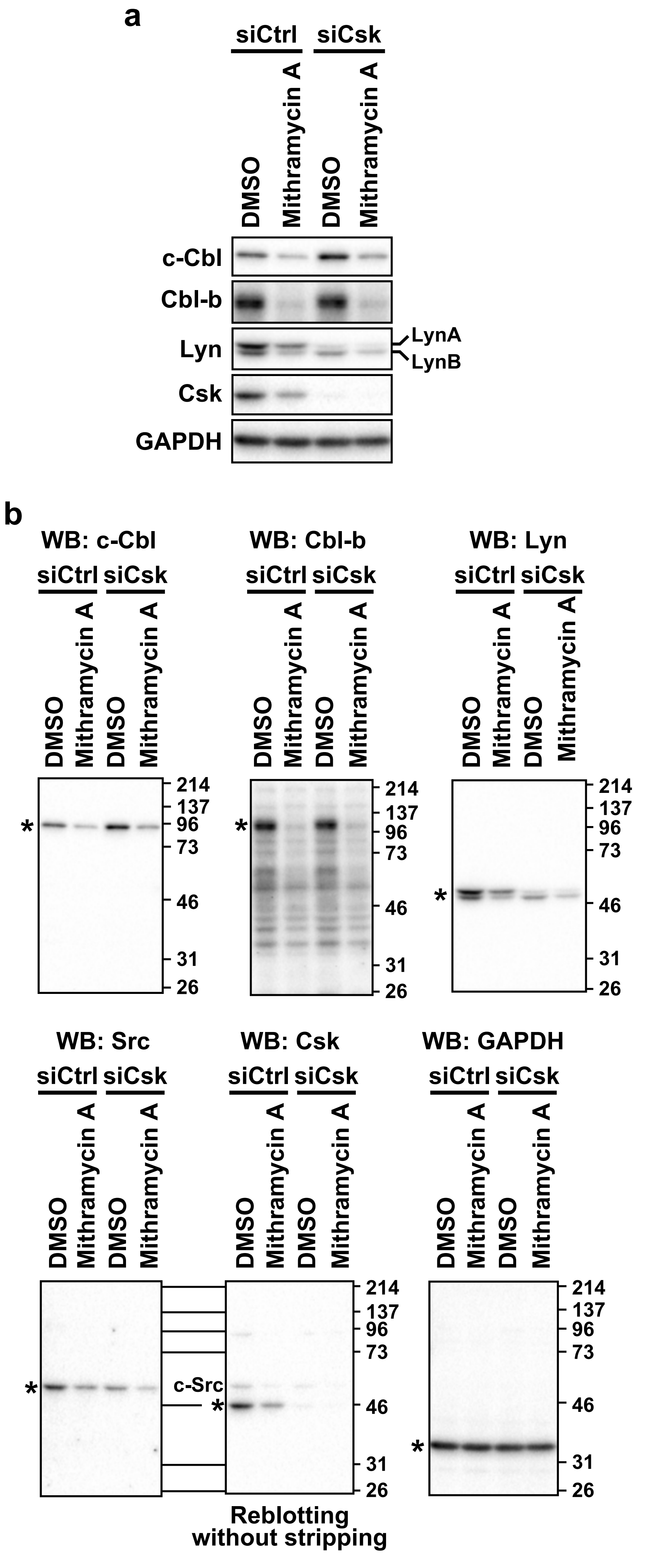
a
siCtrl
siCsk
DMSO
Mithramycin A
DMSO
Mithramycin A
c-Cbl
Cbl-b
Lyn
LynA
LynB
Csk
GAPDH
b
WB: c-Cbl
WB: Cbl-b
WB: Lyn
WB: Src
WB: Csk
WB: GAPDH
siCtrl
siCsk
DMSO
Mithramycin A
DMSO
Mithramycin A
214
137
96
73
46
31
26
*
c-Src
Reblotting
without stripping

Figure S4

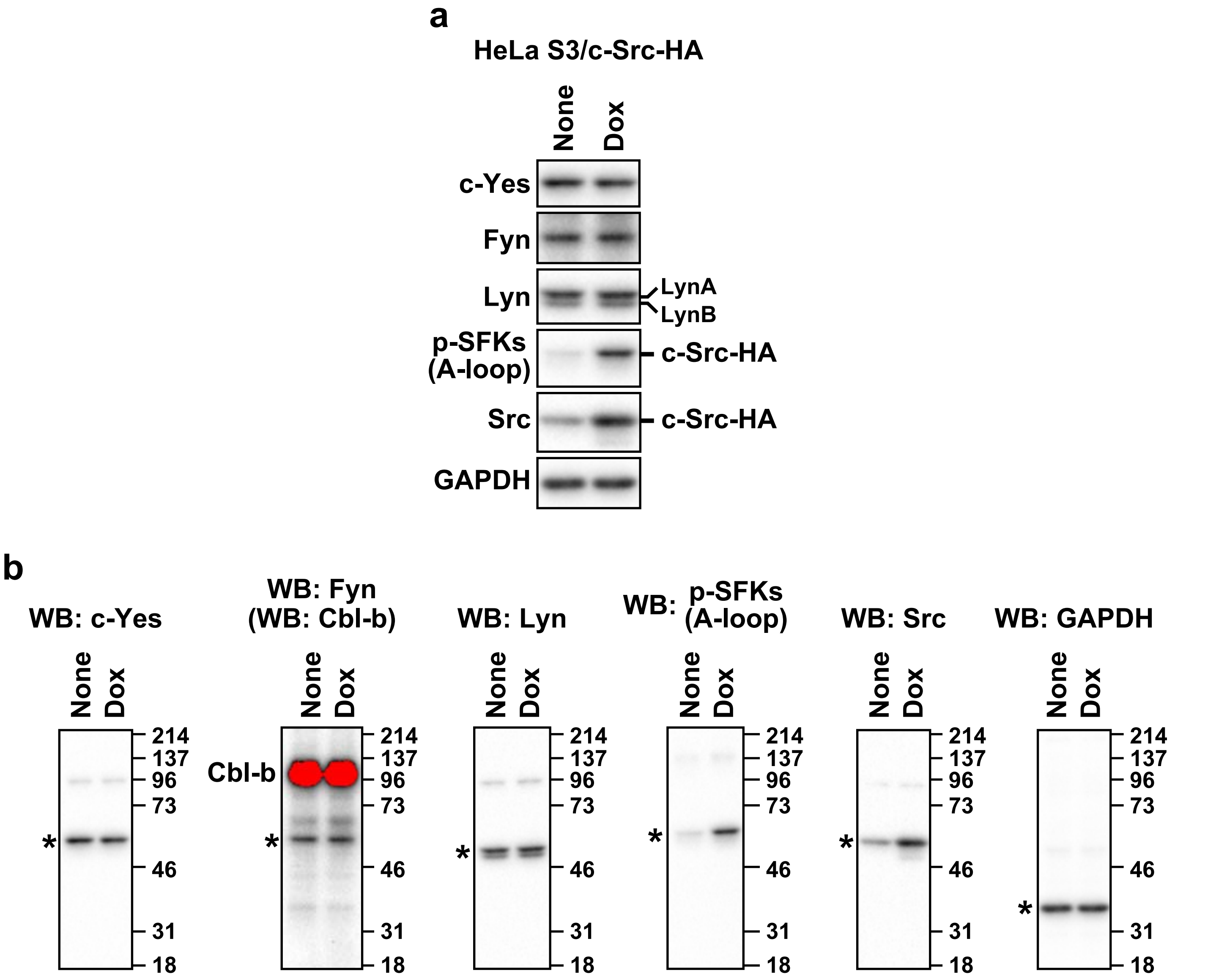
a
HeLa S3/c-Src-HA
None
Dox
c-Yes
Fyn
Lyn
LynA
LynB
p-SFKs (A-loop)
c-Src-HA
Src
c-Src-HA
GAPDH
b
WB: c-Yes
WB: Fyn (WB: Cbl-b)
Cbl-b
WB: Lyn
WB: p-SFKs (A-loop)
WB: Src
WB: GAPDH
None
Dox
214
137
96
73
46
31
18
*

Figure S5

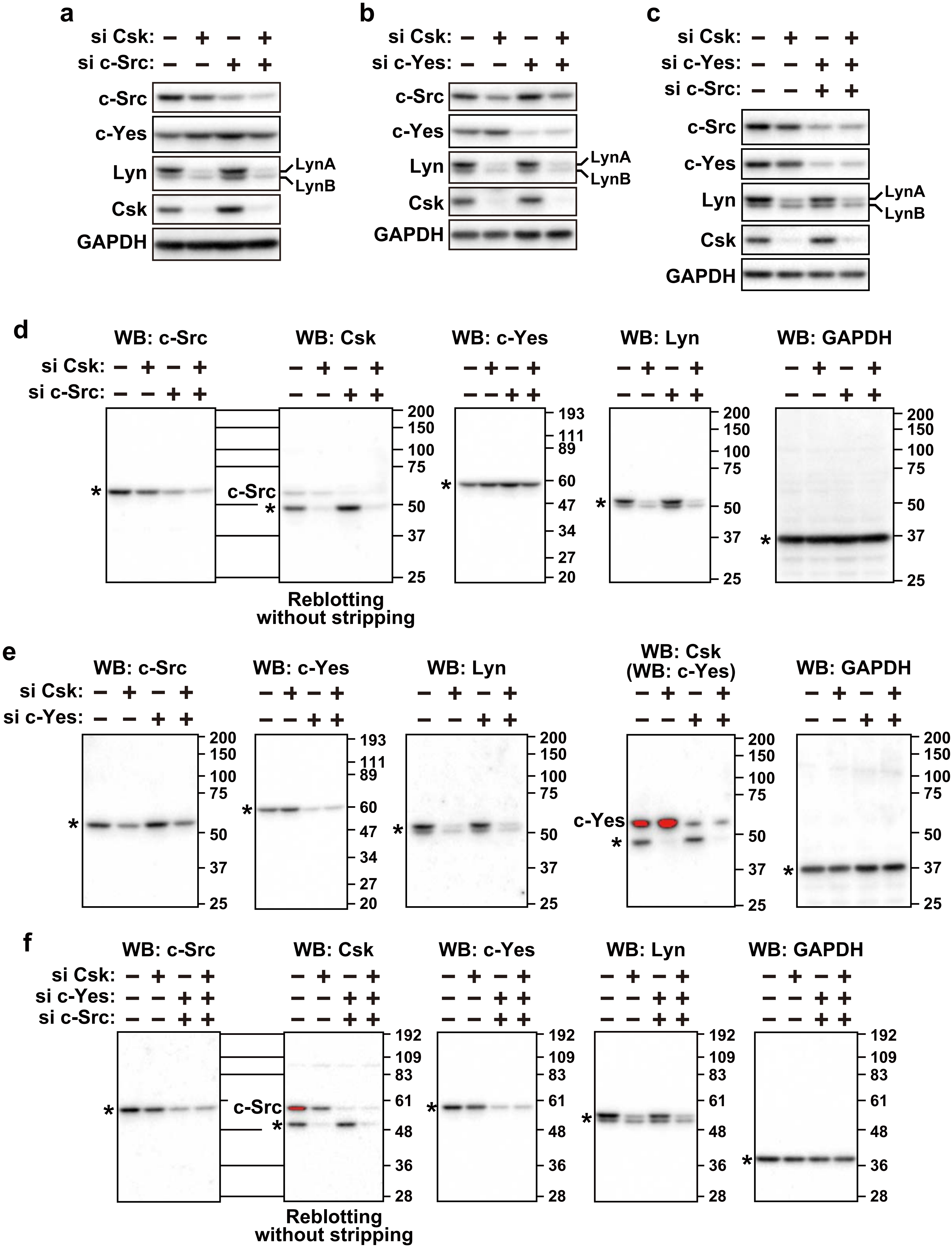
a
si Csk: – + – +
si c-Src: – – + +
c-Src
c-Yes
Lyn
LynA
LynB
Csk
GAPDH
b
si Csk: – + – +
si c-Yes: – – + +
c-Src
c-Yes
Lyn
LynA
LynB
Csk
GAPDH
c
si Csk: – + – +
si c-Yes: – – + +
si c-Src: – – + +
c-Src
c-Yes
Lyn
LynA
LynB
Csk
GAPDH
d
WB: c-Src
WB: Csk
WB: c-Yes
WB: Lyn
WB: GAPDH
si Csk:
si c-Src:
c-Src
Reblotting without stripping
e
WB: c-Src
WB: c-Yes
WB: Lyn
WB: Csk (WB: c-Yes)
WB: GAPDH
si Csk:
si c-Yes:
c-Yes
f
WB: c-Src
WB: Csk
WB: c-Yes
WB: Lyn
WB: GAPDH
si Csk:
si c-Yes:
si c-Src:
c-Src
Reblotting without stripping

Figure S6

a

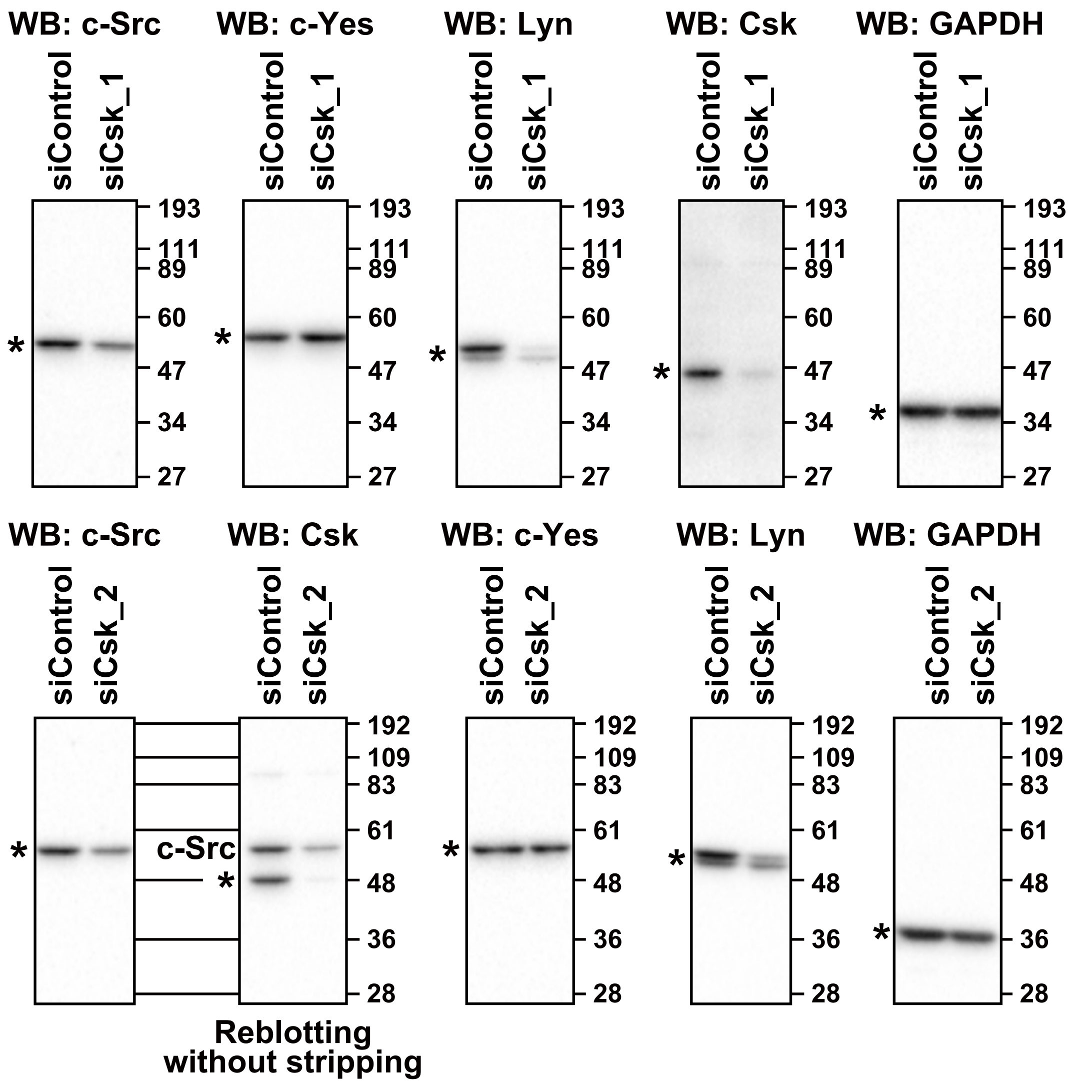

WB: c-Src
WB: c-Yes
WB: Lyn
WB: Csk
WB: GAPDH
siControl
siCsk_1
193
111
89
60
47
34
27
*
WB: c-Src
WB: Csk
WB: c-Yes
WB: Lyn
WB: GAPDH
siControl
siCsk_2
192
109
83
61
48
36
28
c-Src
*
Reblotting
without stripping

b

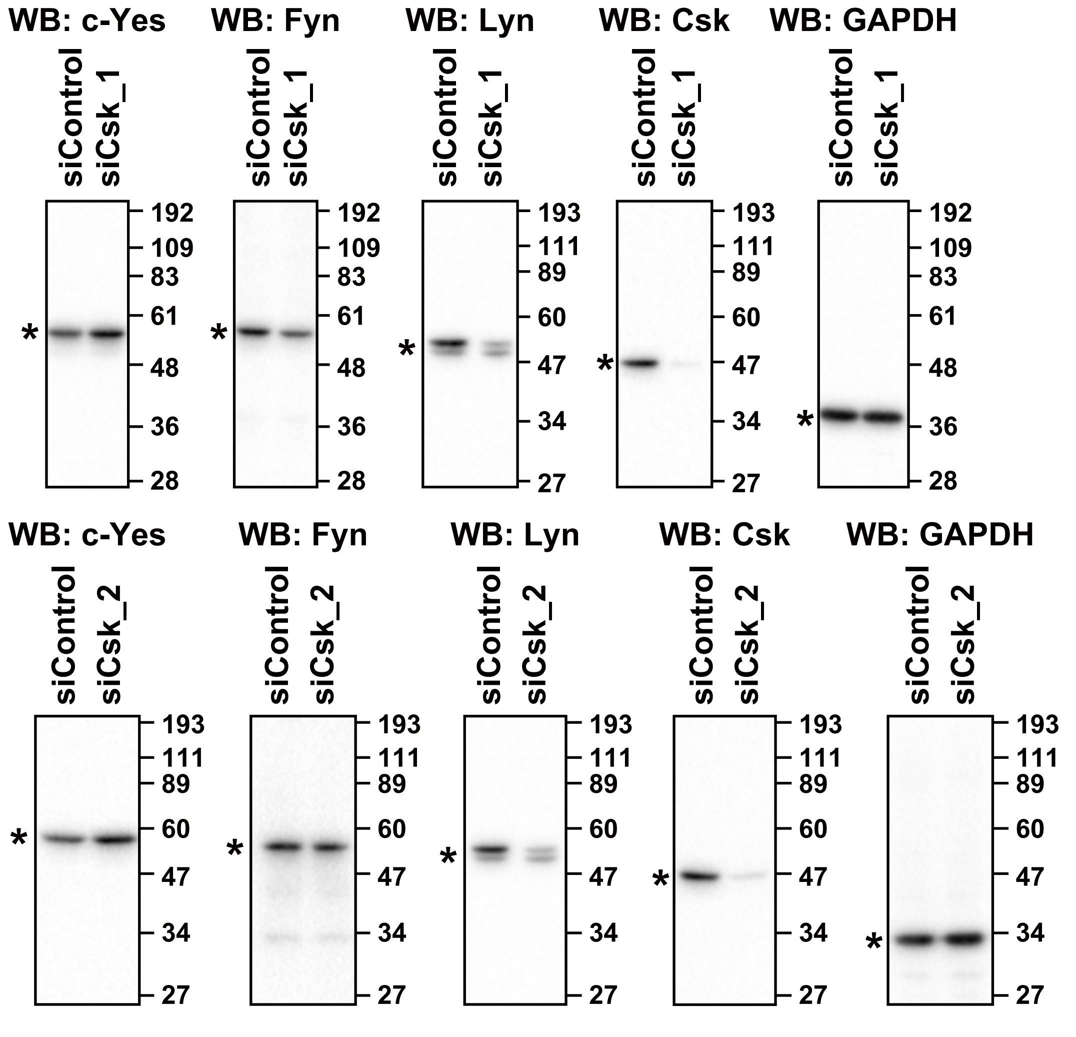

WB: c-Yes
WB: Fyn
WB: Lyn
WB: Csk
WB: GAPDH
siControl
siCsk_1
192
109
83
61
48
36
28
193
111
89
60
47
34
27
*
WB: c-Yes
WB: Fyn
WB: Lyn
WB: Csk
WB: GAPDH
siControl
siCsk_2
193
111
89
60
47
34
27

Figure S7

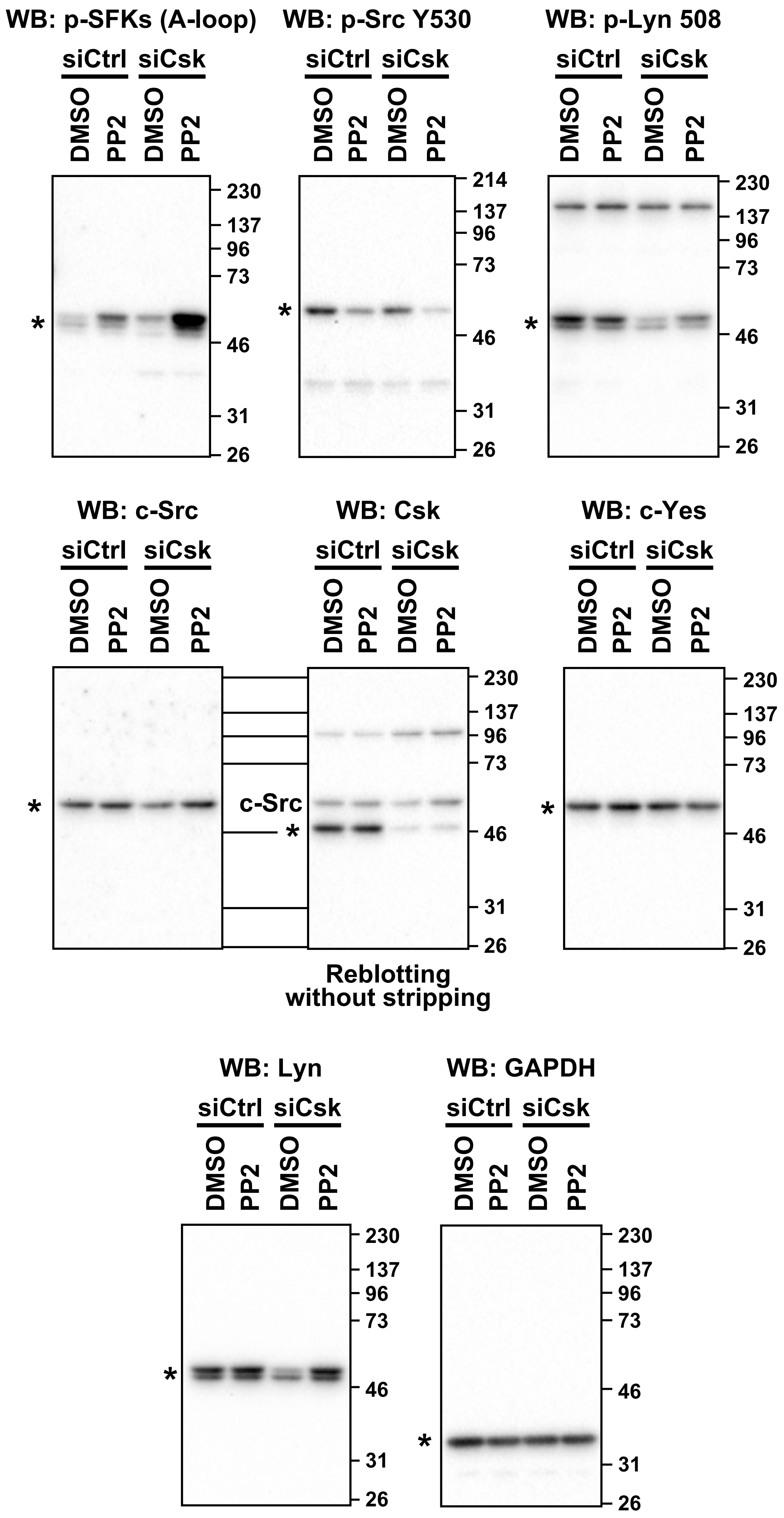

WB: p-SFKs (A-loop)
WB: p-Src Y530
WB: p-Lyn 508
siCtrl
siCsk
DMSO
PP2
230
214
137
96
73
46
31
26
*
WB: c-Src
WB: Csk
WB: c-Yes
c-Src
Reblotting
without stripping
WB: Lyn
WB: GAPDH

Figure S8

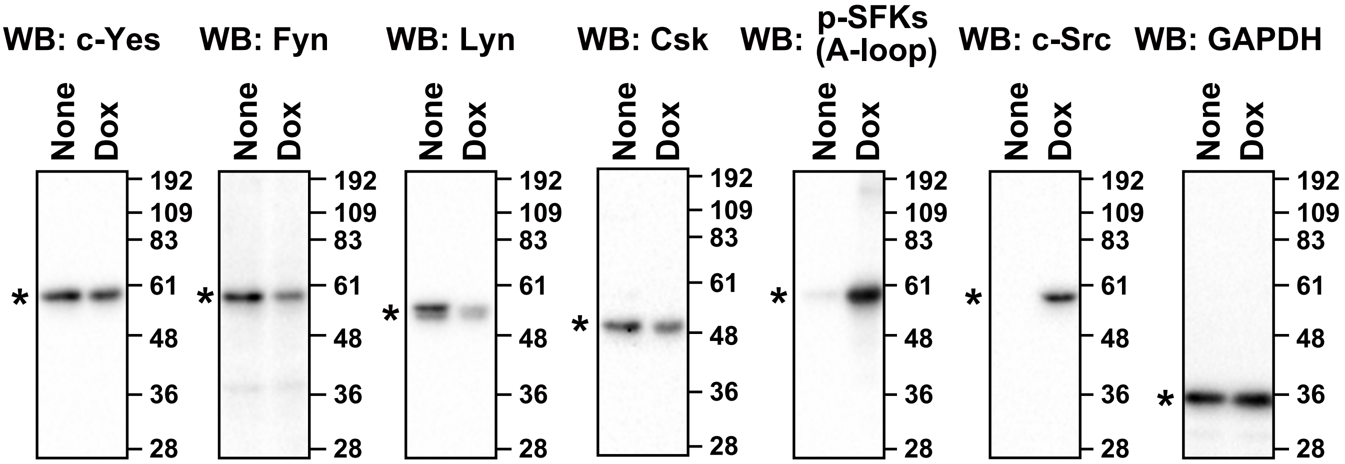
WB: c-Yes
WB: Fyn
WB: Lyn
WB: Csk
WB: p-SFKs (A-loop)
WB: c-Src
WB: GAPDH
None
Dox
192
109
83
61
48
36
28
*

Figure S9

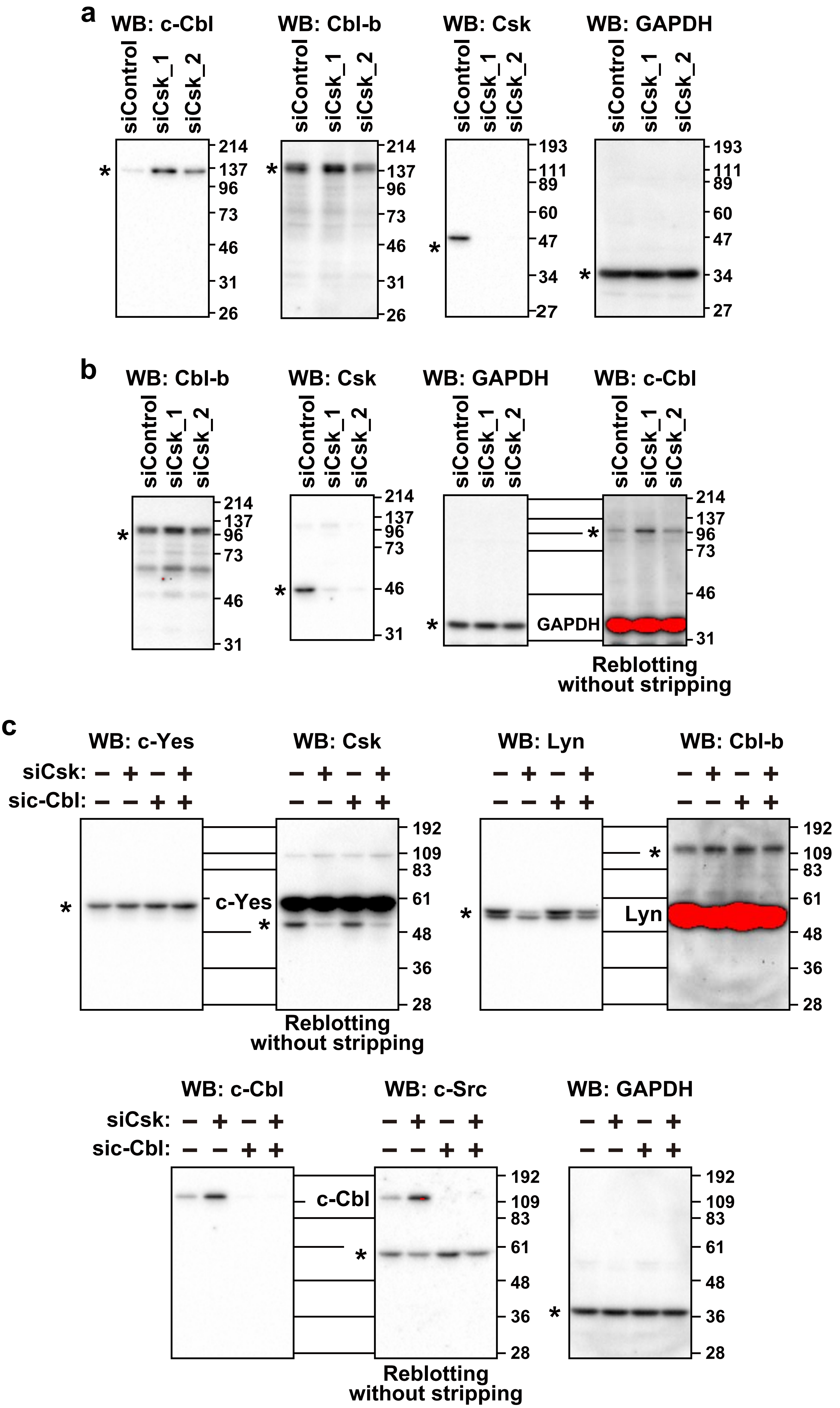
a
WB: c-Cbl
WB: Cbl-b
WB: Csk
WB: GAPDH
siControl
siCsk_1
siCsk_2
214
137
96
73
46
31
26
193
111
89
60
47
34
27
b
WB: Cbl-b
WB: Csk
WB: GAPDH
WB: c-Cbl
GAPDH
Reblotting without stripping
c
WB: c-Yes
WB: Csk
WB: Lyn
WB: Cbl-b
siCsk:
sic-Cbl:
c-Yes
Lyn
192
109
83
61
48
36
28
Reblotting without stripping
WB: c-Cbl
WB: c-Src
WB: GAPDH
c-Cbl
Reblotting without stripping

Figure S10-1

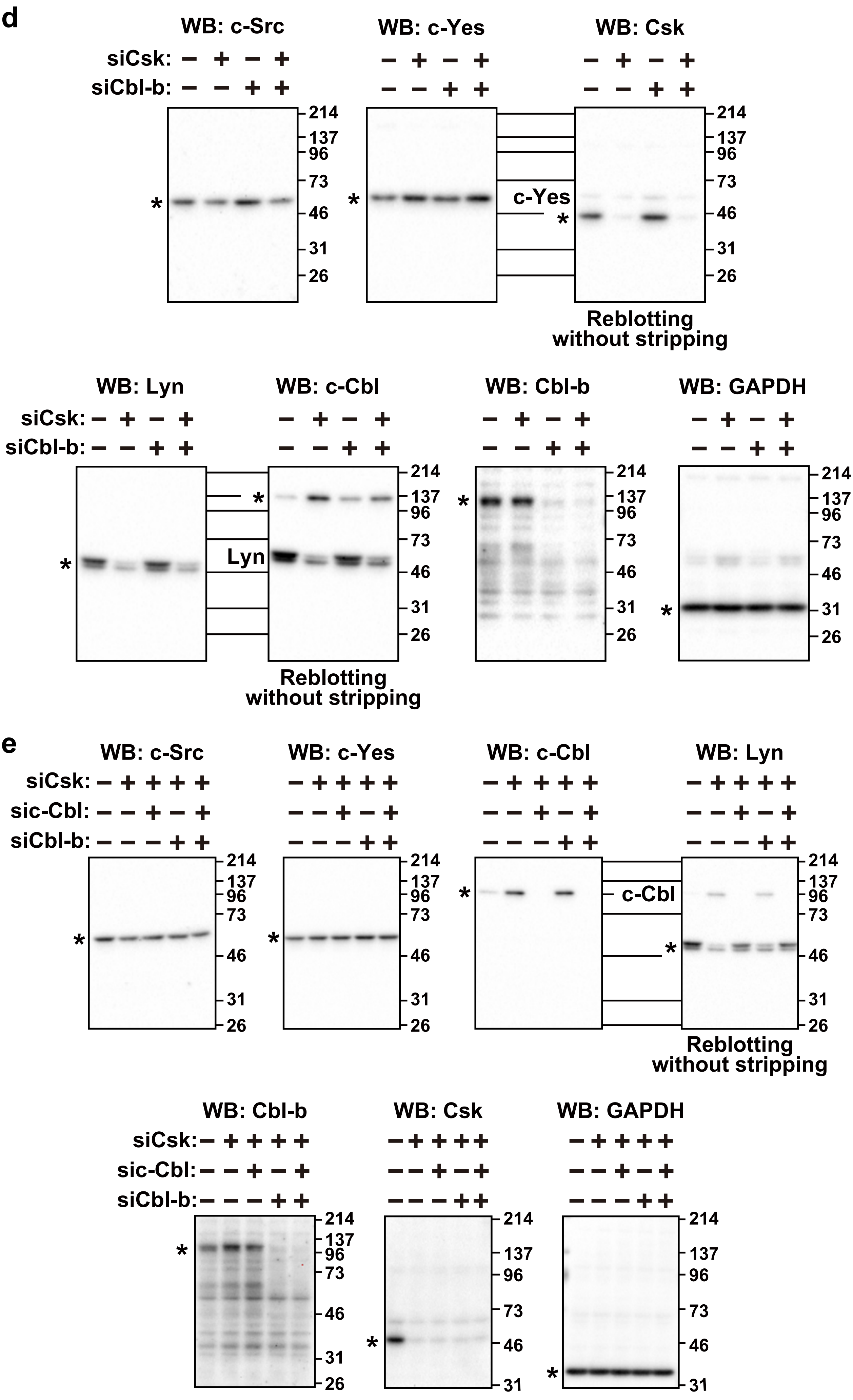
d
WB: c-Src
WB: c-Yes
WB: Csk
siCsk: − + − +
siCbl-b: − − + +
214
137
96
73
46
31
26
c-Yes
*
Reblotting without stripping
WB: Lyn
WB: c-Cbl
WB: Cbl-b
WB: GAPDH
siCsk: − + − +
siCbl-b: − − + +
Lyn
Reblotting without stripping
e
WB: c-Src
WB: c-Yes
WB: c-Cbl
WB: Lyn
siCsk: − + + + +
sic-Cbl: − − + − +
siCbl-b: − − − + +
c-Cbl
Reblotting without stripping
WB: Cbl-b
WB: Csk
WB: GAPDH
siCsk: − + + + +
sic-Cbl: − − + − +
siCbl-b: − − − + +

Figure S10-2

f

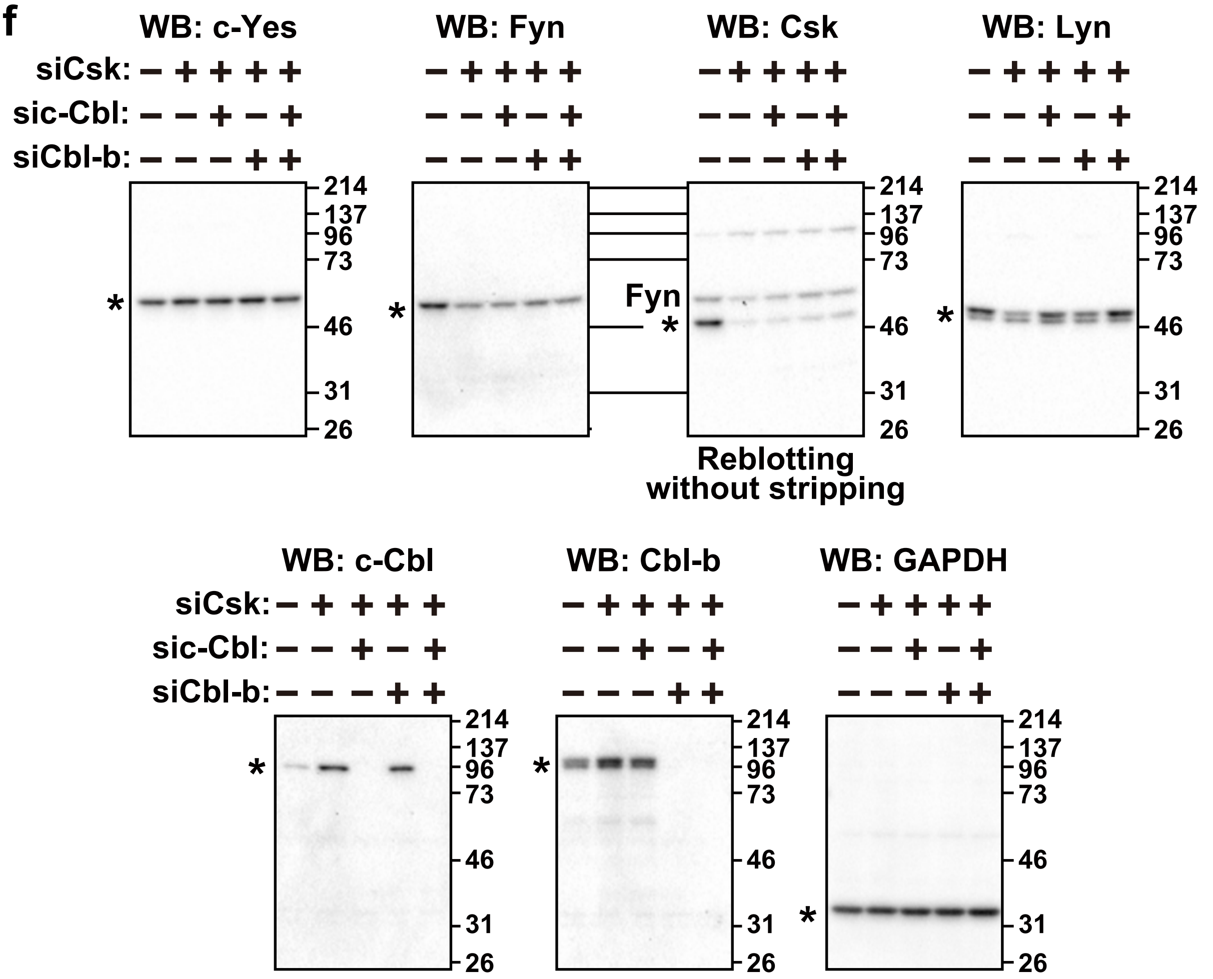

Figure S10-3

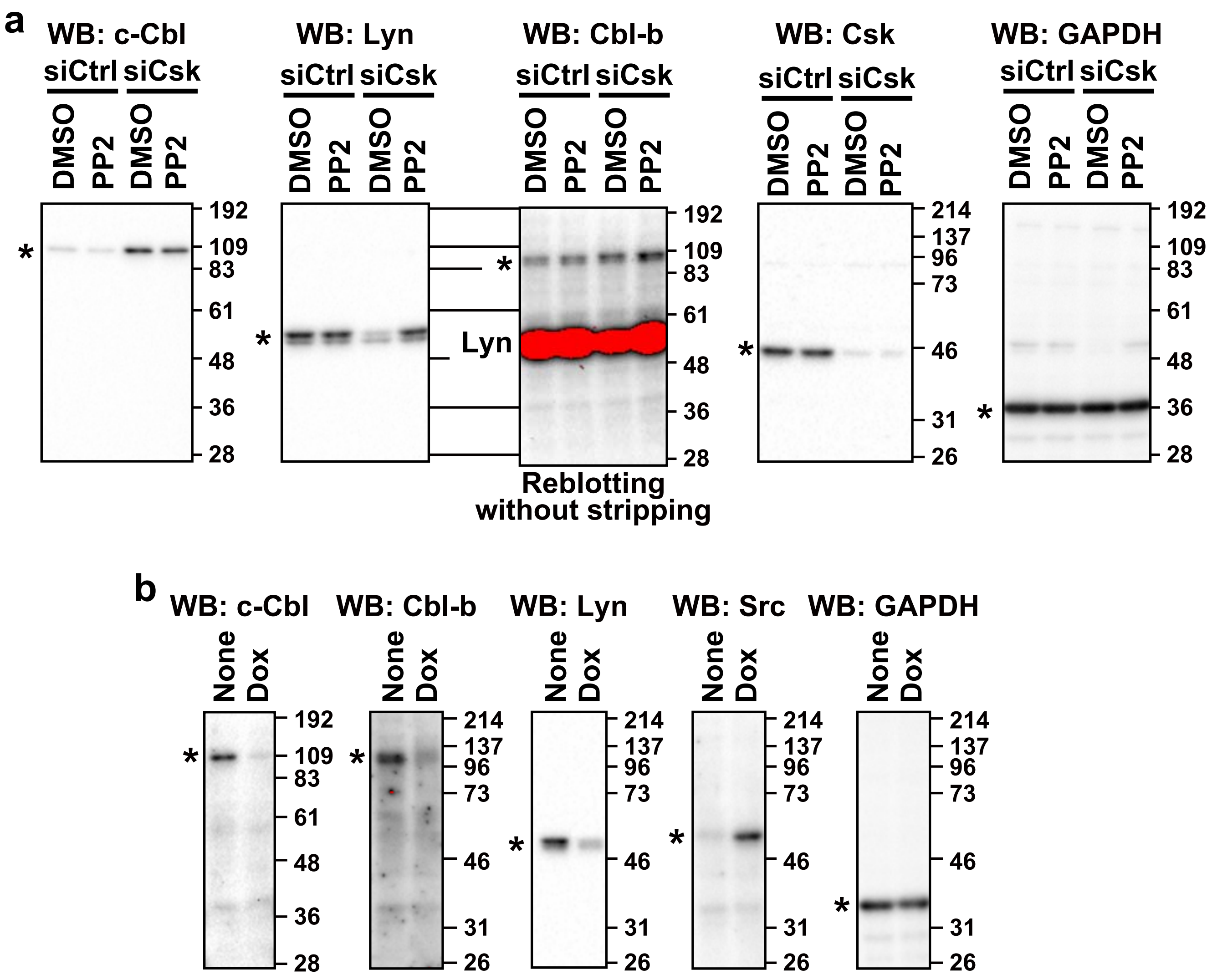
a
WB: c-Cbl
WB: Lyn
WB: Cbl-b
WB: Csk
WB: GAPDH
siCtrl
siCsk
DMSO
PP2
Lyn
Reblotting without stripping
192
109
83
61
48
36
28
214
137
96
73
46
31
26
b
WB: c-Cbl
WB: Cbl-b
WB: Lyn
WB: Src
WB: GAPDH
None
Dox

Figure S11

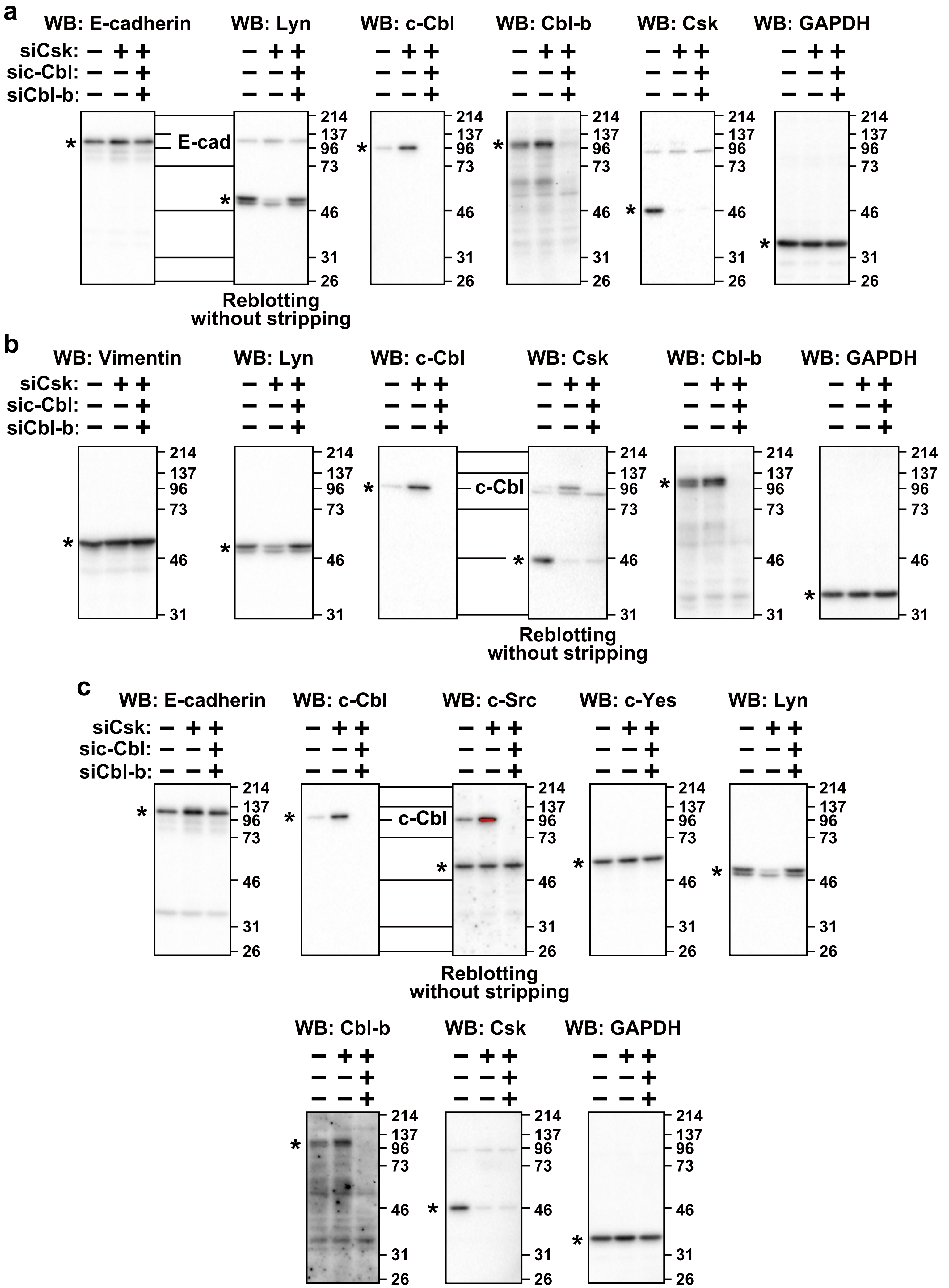
a
WB: E-cadherin
WB: Lyn
WB: c-Cbl
WB: Cbl-b
WB: Csk
WB: GAPDH
siCsk: − + +
sic-Cbl: − − +
siCbl-b: − − +
214
137
96
73
46
31
26
E-cad
Reblotting without stripping
b
WB: Vimentin
WB: Lyn
WB: c-Cbl
WB: Csk
WB: Cbl-b
WB: GAPDH
siCsk: − + +
sic-Cbl: − − +
siCbl-b: − − +
c-Cbl
Reblotting without stripping
c
WB: E-cadherin
WB: c-Cbl
WB: c-Src
WB: c-Yes
WB: Lyn
siCsk: − + +
sic-Cbl: − − +
siCbl-b: − − +
c-Cbl
Reblotting without stripping
WB: Cbl-b
WB: Csk
WB: GAPDH

Figure S12-1

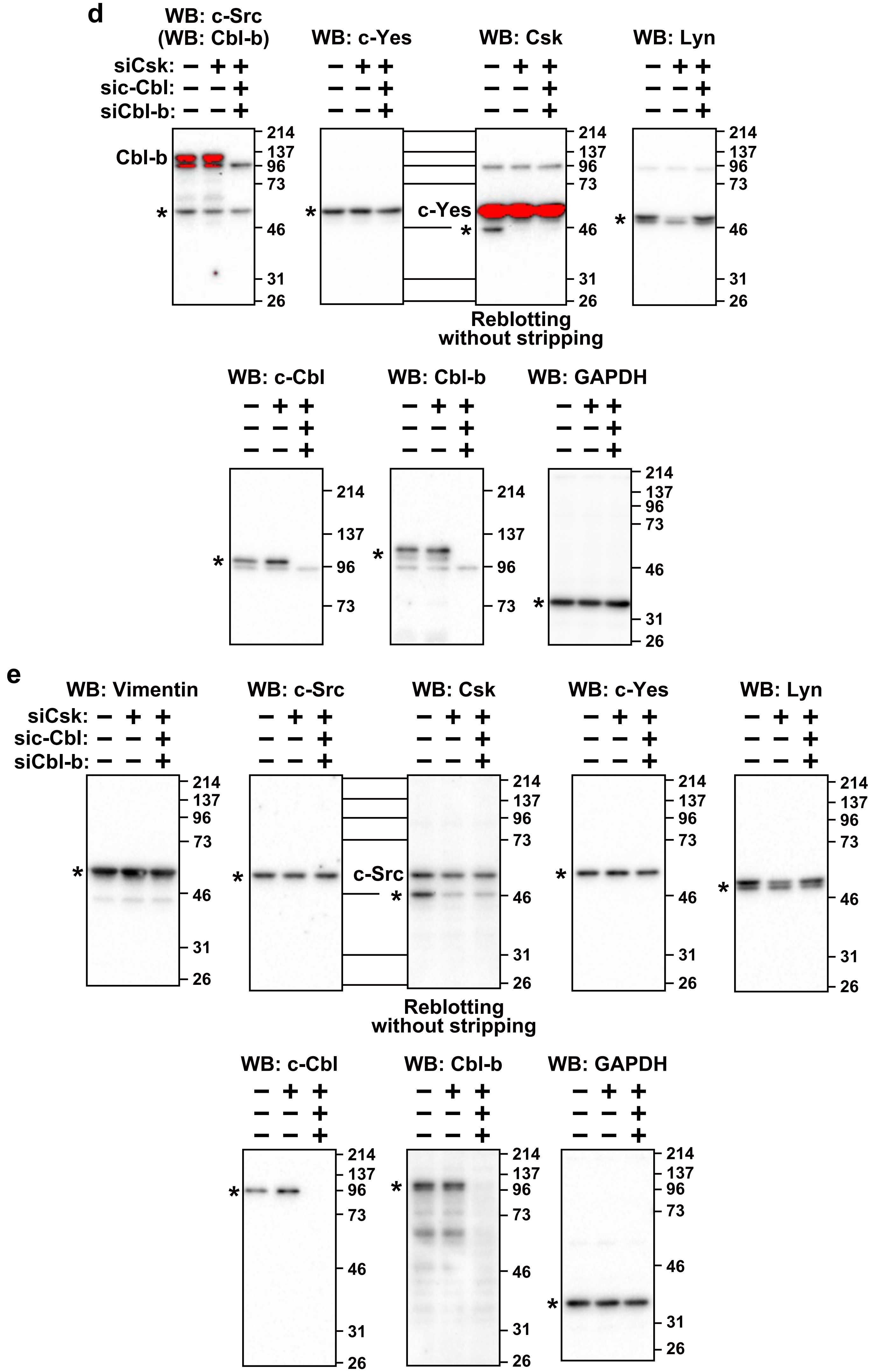
d
WB: c-Src
(WB: Cbl-b)
WB: c-Yes
WB: Csk
WB: Lyn
siCsk:
sic-Cbl:
siCbl-b:
Cbl-b
c-Yes
Reblotting without stripping
WB: c-Cbl
WB: Cbl-b
WB: GAPDH
214
137
96
73
46
31
26
e
WB: Vimentin
WB: c-Src
WB: Csk
WB: c-Yes
WB: Lyn
c-Src
Reblotting without stripping
WB: c-Cbl
WB: Cbl-b
WB: GAPDH

Figure S12-2

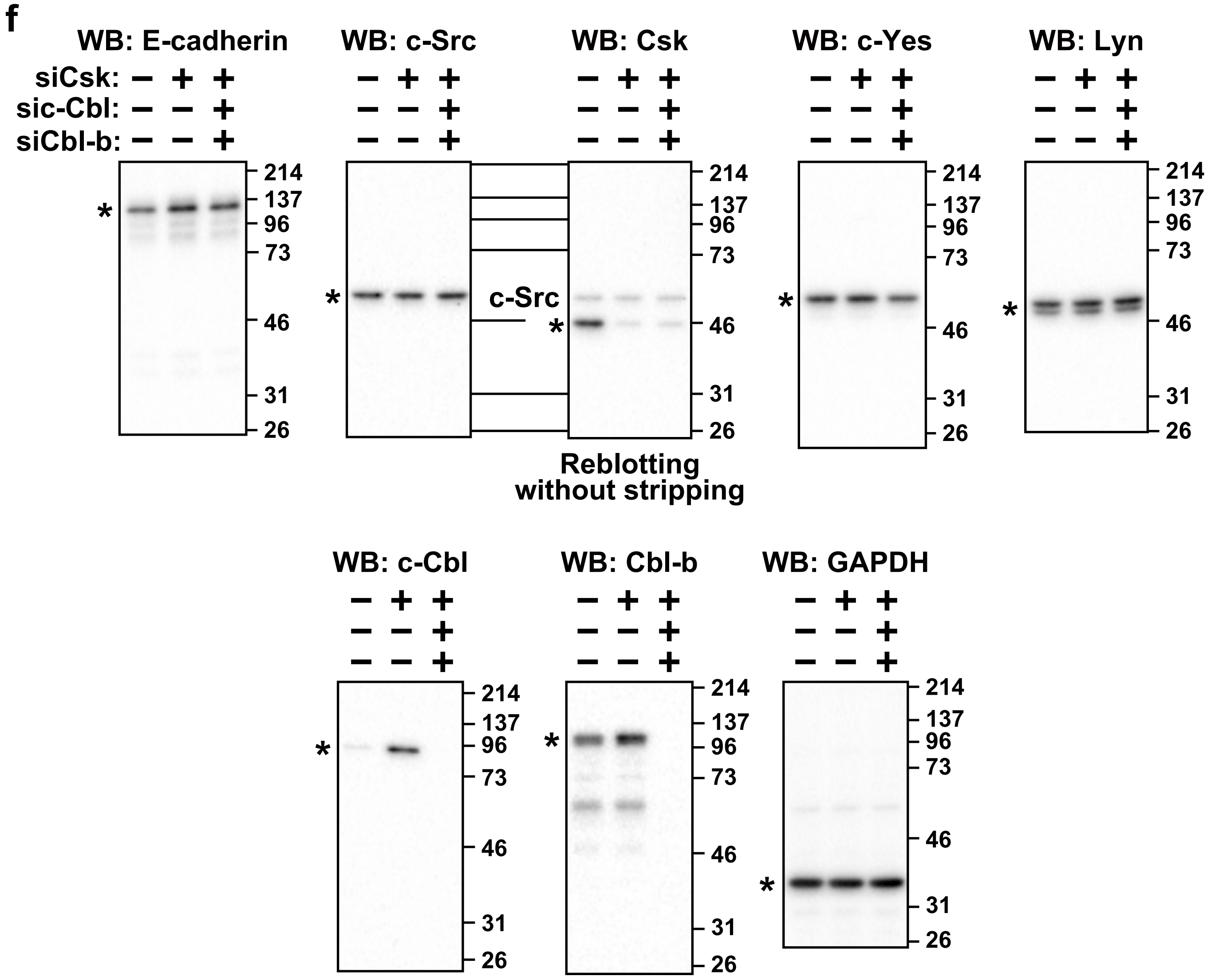
f
WB: E-cadherin
siCsk:
sic-Cbl:
siCbl-b:
214
137
96
73
46
31
26
WB: c-Src
WB: Csk
c-Src
Reblotting
without stripping
WB: c-Yes
WB: Lyn
WB: c-Cbl
WB: Cbl-b
WB: GAPDH

Figure S12-3
